# *Giardia duodenalis* Surface Cysteine Proteases Induce Cleavage of the Intestinal Epithelial Cytoskeletal Protein Villin via Myosin Light Chain Kinase

**DOI:** 10.1371/journal.pone.0136102

**Published:** 2015-09-03

**Authors:** Amol Bhargava, James A. Cotton, Brent R. Dixon, Lashitew Gedamu, Robin M. Yates, Andre G. Buret

**Affiliations:** 1 Department of Biological Sciences, University of Calgary, Calgary, Alberta, Canada; 2 Inflammation Research Network, University of Calgary, Calgary, Alberta, Canada; 3 Host-Parasite Interactions, University of Calgary, Calgary, Alberta, Canada; 4 Bureau of Microbial Hazards, Food Directorate, Health Products and Food Branch, Health Canada, Ottawa, Ontario, Canada; 5 Department of Comparative Biology and Experimental Medicine, University of Calgary, Calgary, Alberta, Canada; 6 Department of Biochemistry and Molecular Biology, University of Calgary, Calgary, Alberta, Canada; Stanford University, UNITED STATES

## Abstract

*Giardia duodenalis* infections are among the most common causes of waterborne diarrhoeal disease worldwide. At the height of infection, *G*. *duodenalis* trophozoites induce multiple pathophysiological processes within intestinal epithelial cells that contribute to the development of diarrhoeal disease. To date, our understanding of pathophysiological processes in giardiasis remains incompletely understood. The present study reveals a previously unappreciated role for *G*. *duodenalis* cathepsin cysteine proteases in intestinal epithelial pathophysiological processes that occur during giardiasis. Experiments first established that *Giardia* trophozoites indeed produce cathepsin B and L in strain-dependent fashion. Co-incubation of *G*. *duodenalis* with human enterocytes enhanced cathepsin production by Assemblage A (NF and S2 isolates) trophozoites, but not when epithelial cells were exposed to Assemblage B (GSM isolate) trophozoites. Direct contact between *G*. *duodenalis* parasites and human intestinal epithelial monolayers resulted in the degradation and redistribution of the intestinal epithelial cytoskeletal protein villin; these effects were abolished when parasite cathepsin cysteine proteases were inhibited. Interestingly, inhibition of parasite proteases did not prevent degradation of the intestinal tight junction-associated protein zonula occludens 1 (ZO-1), suggesting that *G*. *duodenalis* induces multiple pathophysiological processes within intestinal epithelial cells. Finally, this study demonstrates that *G*. *duodenalis*-mediated disruption of villin is, at least, in part dependent on activation of myosin light chain kinase (MLCK). Taken together, this study indicates a novel role for parasite cathepsin cysteine proteases in the pathophysiology of *G*. *duodenalis* infections.

## Introduction

*Giardia duodenalis* (syn. *G*. *intestinalis*, *G*. *lamblia*) is a non-invasive protozoan parasite of the upper small intestines of mammals, including humans. This parasite is a common cause of waterborne diarrhoeal disease worldwide, and is estimated to infect over 280 million individuals annually with 20,000 cases reported per year in the US alone [1, 2]. Importantly, infection has been shown to cause stunting and failure to thrive in young children and in food-producing animals [3–6]. Furthermore, *G*. *duodenalis* infections can also result in the development of extraintestinal or post-infectious complications [7, 8]. Due to the high burden of *G*. *duodenalis*-related illness in the developing world, its impairment on development and socioeconomic improvements, and its close association with poverty, this parasite has been included on the World Health Organization’s (WHO) Neglected Diseases Initiative since 2006 [9]. Therefore, the impact that *G*. *duodenalis* has on society warrants a better understanding of the disease pathophysiology. To date, it has been established that *G*. *duodenalis* causes increased rates of enterocyte apoptosis, intestinal epithelial barrier dysfunction, shortening of small intestinal brush border microvilli in a CD8+ lymphocyte dependent manner, anion hypersecretion, and increased rates of intestinal transit (reviewed in [10]). Furthermore, *G*. *duodenalis* is divided into eight distinct genetic Assemblages, designated A through H, whereby only Assemblages A and B isolates are infective to humans [11, 12]. The idea that *G*. *duodenalis* Assemblages A and B are distinct species is frequently debated [13, 14], and it remains to be determined whether pathogenicity, or the parasite’s immuno-modulating capabilities [15], are assemblage- or isolate-dependent.

Only a small number of *G*. *duodenalis* virulence factors have been identified. Of these, the best characterized are ventral adhesive disc proteins and surface lectins that ensure attachment, the four pairs of flagella that confer re-colonization and movement, and variant surface proteins (VSPs) that evade host IgA-directed clearance [1, 15, 16]. *G*. *duodenalis* also produces an arginine deiminase that prevents intestinal epithelial cells (IECs) from utilizing arginine, thereby impairing intestinal epithelial proliferation and nitric oxide production [17–20]. Although the above-mentioned factors may indirectly contribute to host disease, parasite products directly involved in *G*. *duodenalis-*mediated pathogenesis remain largely unknown. Cathepsin cysteine proteases contain an active site cysteine and histidine residue, and are categorized as clan CA cysteine proteases; in addition, these proteases are further subdivided into cathepsin B (catB) or cathepsin L (catL) superfamilies (reviewed in [21]). Cathepsin-like cysteine proteases are critical to the pathogenesis and pathophysiology of several protozoan parasites (reviewed in ([22, 23]), including *Leishmania donovani* and *Entamoeba histolytica* [24, 25]. The *G*. *duodenalis* genome contains genes for numerous catB- and catL-like cysteine proteases [26, 27] and cysteine protease activity has been reported in *G*. *duodenalis* cultures [28–31]. However, these proteases remain incompletely characterized and their role in giardiasis remains obscure. Thus far, it has been established that cathepsin-like cysteine proteases are required for trophozoite encystation and excystation [32, 33]. Moreover, recent reports demonstrated that *G*. *duodenalis* catB-like cysteine proteases degrade interleukin-8 (CXCL8), leading to attenuated CXCL8-induced neutrophil chemotaxis [34]. However, the effect of these cathepsin-like cysteine proteases on live enterocytes has yet to be evaluated. A better understanding of such proteases in the initiation of disease at the epithelial enterocyte level will further clarify *G*. *duodenalis* pathogenesis, and help identify specific parasitic proteases that may become therapeutic targets [35].

As *G*. *duodenalis* trophozoites are non-invasive, the intestinal epithelium represents a primary point of a contact between host and parasite. This layer is comprised of a single layer of IECs that separate the external environment of the intestinal lumen from underlying host tissues (reviewed in [36]). As such, IECs and their constituent proteins represent an ideal target for *G*. *duodenalis* parasite factors. Indeed, previous work has demonstrated that *G*. *duodenalis* trophozoites induce various pathophysiological processes within IECs [37–40]; however, specific parasite factors have yet to be identified.

Villin is a unique cytoskeletal protein expressed in gastrointestinal, renal, and urogenital epithelial cells. Within the gut, villin is primarily expressed in the microvilli of IEC where it provides an essential function in regulating the organization of epithelial brush border microfilaments during periods of physiological stress [41]. This protein is capable of polymerizing and depolymerizing actin, via its ability to cap, sever, nucleate and bundle actin filaments [42]. *Giardia* is known to shorten epithelial brush border microvilli, thereby contributing to malabsorptive diarrhea [38, 43, 44]. Research has also demonstrated that villin is a pro-survival factor, as its overexpression results in delayed epithelial apoptosis *in vitro*, and its deletion *in vivo* enhances susceptibility to dextran sodium sulfate (DSS)-induced colitis due to increased rates of IEC apoptosis [45]. In its pathogenic cascade, *Giardia* has been found to cause epithelial apoptosis [38, 46]. In addition, villin has been shown to participate in IEC migration and wound healing [42, 47–50]. Collectively, these results demonstrate that villin is a critical homeostatic protein with multiple functions that extend beyond the maintenance of IEC microvilli. The multifunctional purpose of this protein, in addition to its close relationship with F-actin, warrants further research on its interaction with *G*. *duodenalis*. Indeed, previous research has demonstrated that *G*. *duodenalis* trophozoites disrupt intestinal epithelial F-actin [39]. Moreover, remodelling of intestinal epithelial villin has been observed during late stages of *G*. *duodenalis* infection *in vivo* via CD4+ and CD8+ immune responses [51]. However, it remains to be determined whether *G*. *duodenalis* parasite products directly target villin. The objectives of this study were to characterize cysteine protease activity in *G*. *duodenalis* trophozoites during co-incubations with IECs, and to determine whether there is a role for these proteases in intestinal epithelial cytoskeletal villin breakdown and disruption.

## Materials and Methods

### Reagents

Formononetin, the broad-spectrum clan CA cysteine protease inhibitor (2S,3S)-*trans*-Epoxysuccinyl-L-leucylamido-3-methylbutane ethyl ester (E64d) [52], and the myosin light chain kinase inhibitor 1-(5-Chloronaphthalene-1-sulfonyl)-1H-hexahydro-1,4-diazepine hydrochloride (ML-9) were purchased from Sigma-Aldrich (Oakville, ON, Canada). The catB/L fluorogenic substrate Benzyloxycarbonyl-L-Phenylalanyl-L-Arginine 4-Methyl-Coumaryl-7-Amide (ZFR-AMC) [53], the catB-selective fluorogenic substrate Benzyloxycarbonyl-L-Arginine-L-Arginine 4-Methyl-Coumaryl-7-Amide (ZRR-AMC) [54], and the catB-selective inhibitor L-3-trans-(Propylcarbamoyl)Oxirane-2-Carbonyl)-L-Isoleucyl-L-Proline Methyl Ester (Ca-074Me) [55] were purchased from Peptides International (Louisville, KY, USA). The caspase-3 inhibitor Z-DEVD-FMK was purchased from EMD Millipore (Billerica, MA). Zonula occludens-1 (ZO-1) anti-mouse monoclonal antibody (1:500) was purchased from Invitrogen Life Technologies. Villin anti-mouse monoclonal antibody (1:500), glyceraldehyde 3-phosphate dehydrogenase (GAPDH) anti-mouse monoclonal, and actin anti-mouse monoclonal antibody (1:500) were purchased from Santa Cruz Biotechnologies (Dallas, TX). Secondary mouse and rabbit antibodies (1:1000) conjugated with horseradish peroxidase (HRP) were purchased from Cell Signaling Technologies (Beverly, MA). Mouse Alexa Fluor 555-conjugated was purchased from Life Technologies (Carlsbad, CA).

### Cell Culture

Previous research has validated the human adenocarcinoma Caco-2 cell line (ATCC HTB-37) as a reliable model for studying giardiasis *in vitro* [34, 39, 56, 57]. Caco-2 cells were grown in Minimum Essential Medium Eagle (MEME) supplemented with 100 μg/ml streptomycin, 100 Units/ml penicillin, 200 mM L-glutamine, 5mM sodium pyruvate (all from Sigma-Aldrich), and 20% heat-inactivated fetal bovine serum (FBS) (VWR, Radnor, PA). The cells were subcultured using 2X Trypsin-EDTA into 6-well plates (Becton Dickinson, Sparks, MD) or Lab-Tek chamber slides (Nalgene Nunc International, Naperville, IL) when flasks were at approximately 80% confluence. The media in the subcultures and flasks was replaced every 2–3 days. The cells were incubated at 37°C, 5% CO_2_, and 96% humidity. Cells were used between passages 22 and 34.

### Parasites

*G*. *duodenalis* NF trophozoites (Assemblage A) were originally obtained following an epidemic of human giardiasis in Newfoundland, Canada [37, 39], *G*. *duodenalis* S2 isolate parasites (Assemblage A) were obtained from a sheep [37, 58], and *G*. *duodenalis* GS/M isolate (Assemblage B) was obtained from the American Type Culture Collection (ATCC 50581) [59]. Trophozoites were cultured axenically in Keister’s modified TY1-S-33 medium [60, 61] supplemented with piperacillin (Sigma-Aldrich). Trophozoites were grown and passaged in 15 ml polystyrene conical tubes (Benton Dickinson Falcon) at 37°C under anaerobic conditions. Experiments were performed when cultures were at peak density.

### *Giardia duodenalis* trophozoite isolation

Confluent tubes of *G*. *duodenalis* trophozoites were collected by cold shock on ice for 30 min. Following this, 15ml tubes were pooled into 50 ml polypropylene tubes, centrifuged at 500*g* for 10 min at 4°C, and resulting supernatants were aspirated. The pellets were resuspended in 10ml of chilled sterile 1X phosphate buffered saline (PBS) (Sigma-Aldrich) and centrifuged at 500*g* for 10 min at 4°C. The resulting pellet was resuspended in Caco-2 growth media, the trophozoites enumerated using a hemocytometer, and their concentration was adjusted to a multiplicity of infection (MOI) of 10 parasites per 1 host cell (MOI of 10:1).

### *Giardia duodenalis* trophozoite DNA extraction

In preparation for DNA extraction, trophozoites were harvested by placing culture tubes on ice for 10 min. Tubes were then gently inverted to mix the contents, and 2 ml of each suspension were pipetted into 15 ml centrifuge tubes (Falcon), and PBS pH 7.4 was added to a final volume of 10 ml. Tubes were centrifuged at 1,000*g* for 5 min. The supernatants were decanted and the pellets were washed twice more by repeating the centrifugation. Trophozoites were suspended in a final volume of 1 ml PBS. Total DNA was extracted from the trophozoites using the DNeasy Tissue Kit (Qiagen Inc., Mississauga, ON), using a modified protocol. Two hundred microliters of the suspended trophozoites were transferred to 1.5 ml microcentrifuge tubes, and lysed overnight at 56°C using 180 μl of lysis buffer and 20 μl of proteinase K (20 mg/ml) supplied with the DNeasy Tissue Kit. The manufacturer’s instructions were then followed to purify the DNA. Nucleic acid was eluted with 100 μl of elution buffer. Positive control, *G*. *duodenalis* cysts (Waterborne, Inc., New Orleans, LA), and negative control, DNase-free water (Sigma-Aldrich Canada Co., Oakville, ON), were also extracted in parallel.

### PCR Protocols

A nested-PCR was performed to amplify fragments of the *Giardia* 16S rRNA gene as described [62]. PCR amplification of fragments of the glutamate dehydrogenase (gdh) gene for *Giardia*, and a restriction fragment length polymorphism (RFLP) assay for genotyping, was performed as described [63].

### DNA Sequencing

DNA sequencing of the products of both 16S rRNA and gdh PCR was performed at the McGill University and Genome Quebec Innovation Centre in Montreal, QC, using a 3730xl DNA Analyser (Applied Biosystems, Foster City, CA). PCR products were purified and bi-directional sequencing was performed using the same primers as the original amplifications. DNA sequences were assembled, edited and aligned using SeqScape v2.5 (ABI). Resulting consensus sequences were then aligned with representative sequence data from *G*. *duodenalis* Assemblages and trimmed to identical lengths of 189 bp or 367 bp for 16S rRNA and gdh genes, respectively, using Bioedit v7.1.3.0 [64]. Consensus sequences were compared to reference sequences in GenBank using NCBI standard nucleotide BLAST (blastn). Phylogenetic trees were generated using Molecular Evolutionary Genetics Analysis (MEGA v5.2.1) (http://www.megasoftware.net/) with a Kimura two parameter model neighbour-joining analysis, with 100 bootstraps and pair-wise deletion.

### *Giardia duodenalis* trophozoite viability

Motility of *G*. *duodenalis* trophozoites was used to assess parasite viability after 2- or 24-hour co-incubation. Subsequently, Caco-2 cell supernatants were collected, vortexed, and 10 μL of cell supernatant was analyzed on a hemocytometer. The ratio of motile, or swimming, trophozoites to total trophozoite counts was assessed as a marker of viability [65].

### *Giardia duodenalis* modulation of intestinal epithelial villin

*G*. *duodenalis* trophozoites (NF, S2, or GS/M) were co-incubated with confluent Caco-2 monolayers at an MOI of 10:1 for 2 or 24 hours. Identical experiments were performed, except trophozoites were initially pre-treated with E-64d (10 μM), Ca-074Me (10 μM), or vehicle control (dimethyl sulfoxide; DMSO) 3 hours prior (see below) to co-incubation with *in vitro* Caco-2 monolayers. Similarly, Caco-2 monolayers were pre-treated with one of E-64d (10 μM), Ca-074Me (10 μM), ML-9 (40 μM), Z-DEVD-FMK (50 μM), or vehicle control (DMSO) prior to co-incubation with *G*. *duodenalis* trophozoites for 2 or 24 hours. In complimentary experiments, *G*. *duodenalis* NF trophozoites were co-incubated with Caco-2 monolayers grown to confluence on the bottom of 12-well plates; in this instance, *G*. *duodenalis* trophozoites were seeded into the top compartment of 0.4 μm transwells to prevent direct contact between *in vitro* monolayers and parasites. Incubation conditions were maintained at 37°C, 5% CO_2_, and 96% humidity for experimental duration. At the end of the incubation period, cell supernatants were collected and processed for cathepsin activity assays (see below), while monolayers were washed once with PBS and subsequently processed for Western blotting analysis (see below).

In parallel experiments, Caco-2 monolayer cellular lysates were co-incubated with *G*. *duodenalis* NF trophozoite sonicates for 2 hours. Briefly, culture media from confluent Caco-2 monolayers was removed, and cells were washed once with PBS. Following this, the PBS was aspirated, the Caco-2 monolayers were collected in 200μL protease inhibitor-free radioimmunoprecipitation assay (RIPA) buffer (1% Igepal, 0.1% SDS, and 0.5% sodium deoxycholate in PBS) in microcentrifuge tubes, and sonicated at level 3 for 5 seconds (550 Versonic Dismembranator, Fisher Scientific). Caco-2 lysates protein concentrations were adjusted to 3mg/ml using the Bradford assay method (BioRad Laboratories, Hercules, CA). At the same time, *G*. *duodenalis* NF trophozoites were adjusted to a concentration of 1x10^7^ trophozoites/ml and sonicated three times at level 4 for 30 seconds on ice (550 Versonic Dismembranator, Fisher Scientific). The resulting Caco-2 lysates and *G*. *duodenalis* trophozoite sonicates were co-incubated for 2 hours in the presence of 10 mM DTT and in the presence or absence of E-64 (200 μM) or Ca-074Me (200 μM). After 2 hours, samples were processed for Western blotting analysis (see below).

### Whole cell protein extraction for Western blotting

At the end of the co-incubation for 2 or 24 hours, trophozoites were removed from the co-culture via three ice-cold PBS washes. Caco-2 monolayers were subsequently collected in RIPA buffer supplemented with a protease inhibitor cocktail tablet (Complete-Mini, Roche Diagnostics, Laval, QC). After a 30 min incubation at 4°C, cellular lysates were sonicated at level 3 for 5 seconds and centrifuged at 10,000*g* for 10 min at 4°C. The resulting supernatant was collected and protein concentrations determined using the Bradford assay method [66]. Protein samples were then normalized to 1.0 mg/ml and then combined at a 1:1 ratio with 2X electrophoresis buffer (100 mM Tris-HCl, pH 6.8, 4% sodium dodecyl sulfate (SDS), 0.2% bromophenol blue, 20% glycerol, 200 mM β-mercaptoethanol) to further dilute the samples to a final concentration of 0.5 mg/ml. The samples were then denatured at 95°C for 3 min and stored at-20°C until further analysis.

### Western blotting

Protein samples were separated via SDS-PAGE (7–12%) and transferred to nitrocellulose membranes (Whatman, Buckinghamshire, England) over 1 hour at 100V. The membranes were blocked using 5% fat-free milk solution in 1X Tris-buffered saline + 0.1% Tween (TBS-T) for 1 hour. Primary antibodies were diluted in the same 5% milk solution and incubated with the membranes overnight at 4°C. After three 15-min washes with TBS-T, HRP-conjugated secondary antibodies, also diluted in 5% milk solution, were added to the membranes for 1 hour at room temperature. The membranes were washed three times with TBS-T for 15 min each and then visualized using ECL-plus chemifluorescence detection system (GE Healthcare, Pittsburgh, PA). ECL-plus was added the membranes for 5 min. The membranes were visualized on ECL film (GE Healthcare). The films were scanned for densitometric analysis using the software ImageJ (http://rsbweb.nih.gov/ij/). The membranes were stripped using 0.5M acetic acid (45 min incubation) and 0.2 M sodium hydroxide (5 min incubation), and re-probed with a GAPDH antibody to ensure equal loading of the gels. In all instances, GAPDH was used as the loading control.

### Inhibition of *G*. *duodenalis* cysteine proteases

Previous research has demonstrated that concentrations of 10μM E-64d or Ca-074Me do not affect *G*. *duodenalis* trophozoite viability, but significantly reduce cathepsin-like cysteine protease activity [34, 67]. Therefore, experiments involving *G*. *duodenalis* trophozoite and Caco-2 monolayer co-incubations were repeated with the administration of either 10μM E-64d, 10 μM Ca-074Me, or vehicle control (DMSO) for 2 or 24 hours. Following this, samples were collected and processed for cathepsin activity assays and Western blotting analysis. In separate experiments, confluent tubes of *G*. *duodenalis* trophozoites were treated with E64d (10μM), Ca-074Me (10μM), or vehicle control (DMSO) for 3 hours. Following this, *G*. *duodenalis* trophozoites were harvested by cold shock on ice for 30 min and then co-incubated with Caco-2 monolayers for 2 or 24 hours. Samples were again collected for cathepsin activity assays and Western blotting analysis.

### Sample extraction for cathepsin activity assays

After co-incubation of *G*. *duodenalis* trophozoites with Caco-2 monolayers (as described above), supernatants, parasites, and IECs were collected and processed for cathepsin activity assays. Cell supernatants were collected, centrifuged at 500*g*, 4°C for 10 min and stored for further use. The supernatant pellet, representing *G*. *duodenalis* trophozoites, was resuspended in ice-cold PBS, centrifuged at 500*g*, 4°C for 10 min, and the trophozoite pellet re-suspended in RIPA buffer; this fraction was then sonicated at level 4 for 30 seconds on ice and centrifuged at 10,000*g*, 4°C for 10 min. A Bradford assay was performed and samples normalized to 1.0 mg/ml. Adherent *G*. *duodenalis* trophozoites were removed from Caco-2 monolayers by modifying a previously described protocol [68]. In short, a sterile 10μM formononetin solution was made in the Caco-2 growth medium, added to Caco-2 monolayers, and then allowed to incubate at 37°C, 5% CO_2_ for 60 min. Following this, the formononetin solution was aspirated, and monolayers were washed with ice-cold PBS three times. Monolayers were then collected and lysed in RIPA buffer not containing a protease inhibitor table, sonicated on level 3 for 5 seconds, and then centrifuged for 10 min at 10,000*g* at 4°C. A Bradford assay was performed on collected Caco-2 lysates and samples were then normalized to 3.0 mg/ml. All samples were stored at-70°C until further use.

### Cathepsin activity assays

Assessment of cathepsin cysteine protease activity was performed via recording the liberation of 7-aminomehtylcoumarin (AMC) from fluorogenic substrates, whereby cathepsin protease activity correlates to an increase in detectable relative light units (RFUs) over time [53, 54]. To assess supernatant cathepsin cysteine protease activity, samples were thawed and incubated at a 1:2 ratio with cathepsin assay buffer (100 mM sodium acetate, 10 mM DTT, 0.1% Triton X-100, 1 mM EDTA, 0.5% DMSO, 200 μM ZFR-AMC or ZRR-AMC). Assessment of intra-trophozoite or intracellular Caco-2 cathepsin cysteine protease activity involved incubating samples at a 1:3 ratio with cathepsin assay buffer. All samples were incubated in 96-well clear, flat-bottom plates and at 37°C for 5 min and subsequently measured kinetically using a microplate reader (SpectraMax M2^e^, Molecular Devices, Sunnyvale, CA) at 37°C with excitation and emission wavelengths of 354nm and 445nm, respectively. Measurements were recorded every 30 seconds for 5 min. For all experiments, cathepsin assay buffer was adjusted to a pH of 7.2 to mimic the luminal pH of the upper small intestine.

### Immunofluorescence

Caco-2 monolayers grown to confluence in Lab-Tek chamber slides (Nalgene Nunc International, Naperville, IL) (8–10 days) were co-incubated for 2 or 24 hours with 1.0x10^7^ *G*. *duodenalis* NF trophozoites pretreated for 3 hours with E64d (10uM) or vehicle control (dimethyl sulfoxide; DMSO) at 37°C, 5% CO_2_. In another set of experiments, Caco-2 monolayers were pre-treated with ML-9 (40μM) for 30 min prior to co-incubation with *G*. *duodenalis* NF trophozoites for 2 or 24 hours. At the end of the co-incubation, the infection medium was aspirated off out of each chamber, followed by two ice-cold PBS washes. To fix/permeabilize the cells, ice-cold methanol was then added to each well and the chamber slides were subsequently incubated at 4°C for 30 min. After two more PBS washes, the cells were blocked with heat inactivated-fetal bovine serum (HI-FBS; VWR) for 15 min at room temperature. Primary antibodies were prepared in a solution of 2% FBS-PBS at appropriate concentrations that was subsequently administered to cells for 1 hour at 37°C. Following two PBS washes, the cells were incubated with fluorescent secondary antibodies (also prepared in 2% FBS-PBS) for 1 hour at 37°C. The cells were subsequently washed twice with PBS. Nuclei were counterstained with 1μM Hoechst fluorescent staining 33258 (Invitrogen Life Technologies) for 30 min at 37°C. The cells were washed once with PBS and the slides were mounted with Aqua Poly/Mount (Polyscience Inc.; Warrington, PA). Micrographs were obtained using a Leica DMR Microscope, with a Retiga 2000R (Q Imaging, BC) at 400x. All images were collected with the same gain and exposure time lengths. Micrographs presented in the Results section are representative images of 2 replicate monolayers from three separate experiments.

### Statistical analysis

All data are representative of at least three separate experiments and expressed as means ± SEM, where applicable. All statistical analyses were performed using the software, Graphpad Prism 6, which ensures normality of data prior to analysis. Comparisons between groups were made using a Student t-test or one-way ANOVA, followed by Tukey’s test for multiple comparison analysis. Statistical significance was established at p<0.05.

## Results

### *Giardia duodenalis* NF and S2 trophozoites are Assemblage A isolates

**Fig 1:**
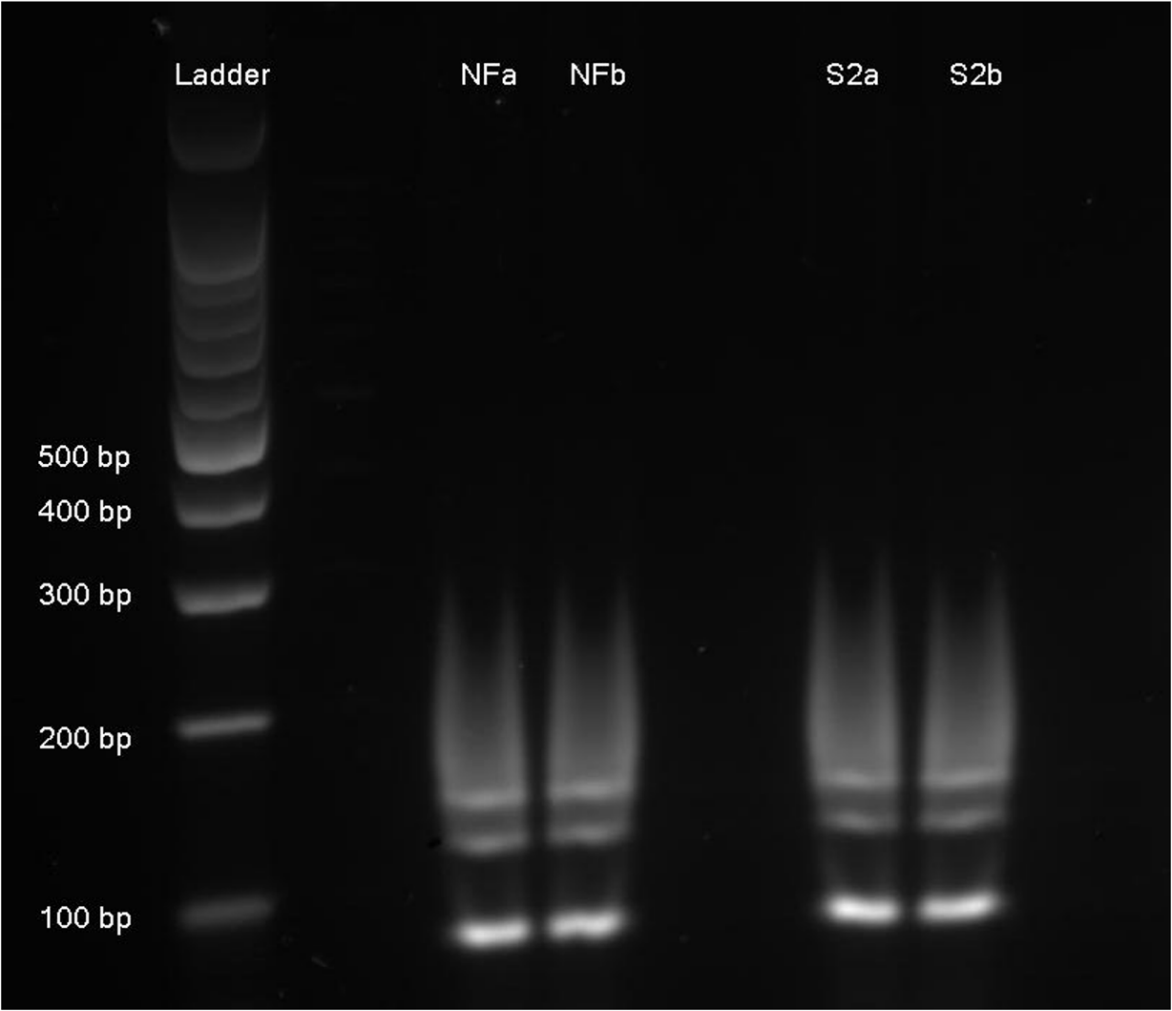
Restriction fragment length polymorphism (RFLP) profile of a fragment of the gdh gene amplified by PCR from NF and S2 isolates of *Giardia duodenalis*. The ladder is a 100 bp molecular weight marker (Promega Corp., Madison, WI). NF and S2 isolates of *Giardia duodenalis* trophozoites were each run in replicate (i.e., NFa and b, S2a and b).

**Fig 2:**
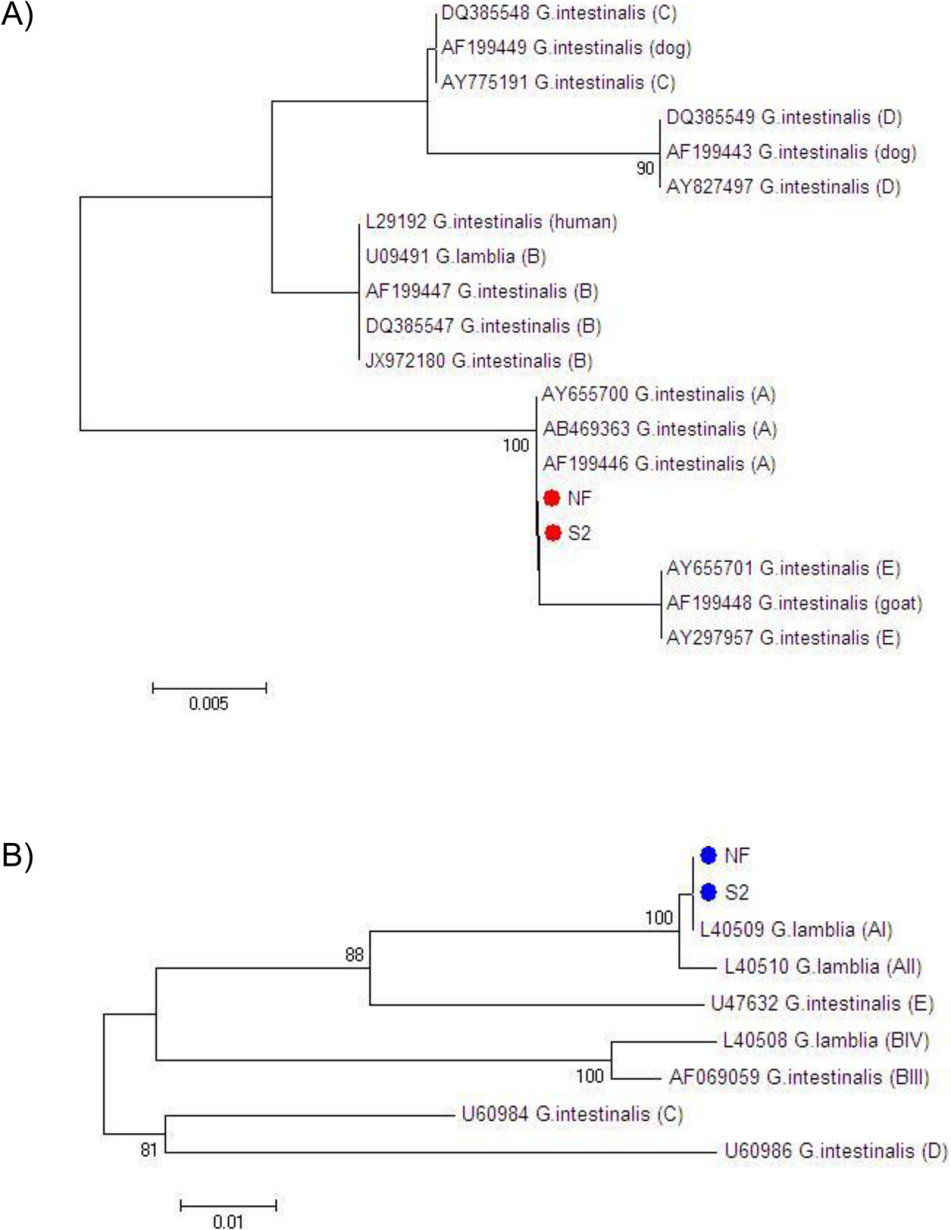
Phylogenetic trees of the NF and S2 isolates of *Giardia duodenalis*. Phylogenetic trees are based on the DNA sequences of amplified fragments of the 16s rRNA (A) and glutamate dehydrogenase (gdh) genes (B).

PCR-RFLP of the glutamate dehydrogenase (gdh) gene revealed identical banding patterns for both NF and S2 *G*. *dudoenalis* isolates, matching the expected banding pattern for *G*. *duodenalis* Assemblage A (Fig 1). DNA sequence data on both 16S rRNA and gdh PCR products demonstrated that both the NF and S2 isolates were 100% homologous to reference sequences of *G*. *duodenalis* Assemblage A. Phylogenetic trees for both loci are shown in Fig 2.

### *Giardia duodenalis* trophozoites contain and release catB and L cysteine proteases

**Fig 3:**
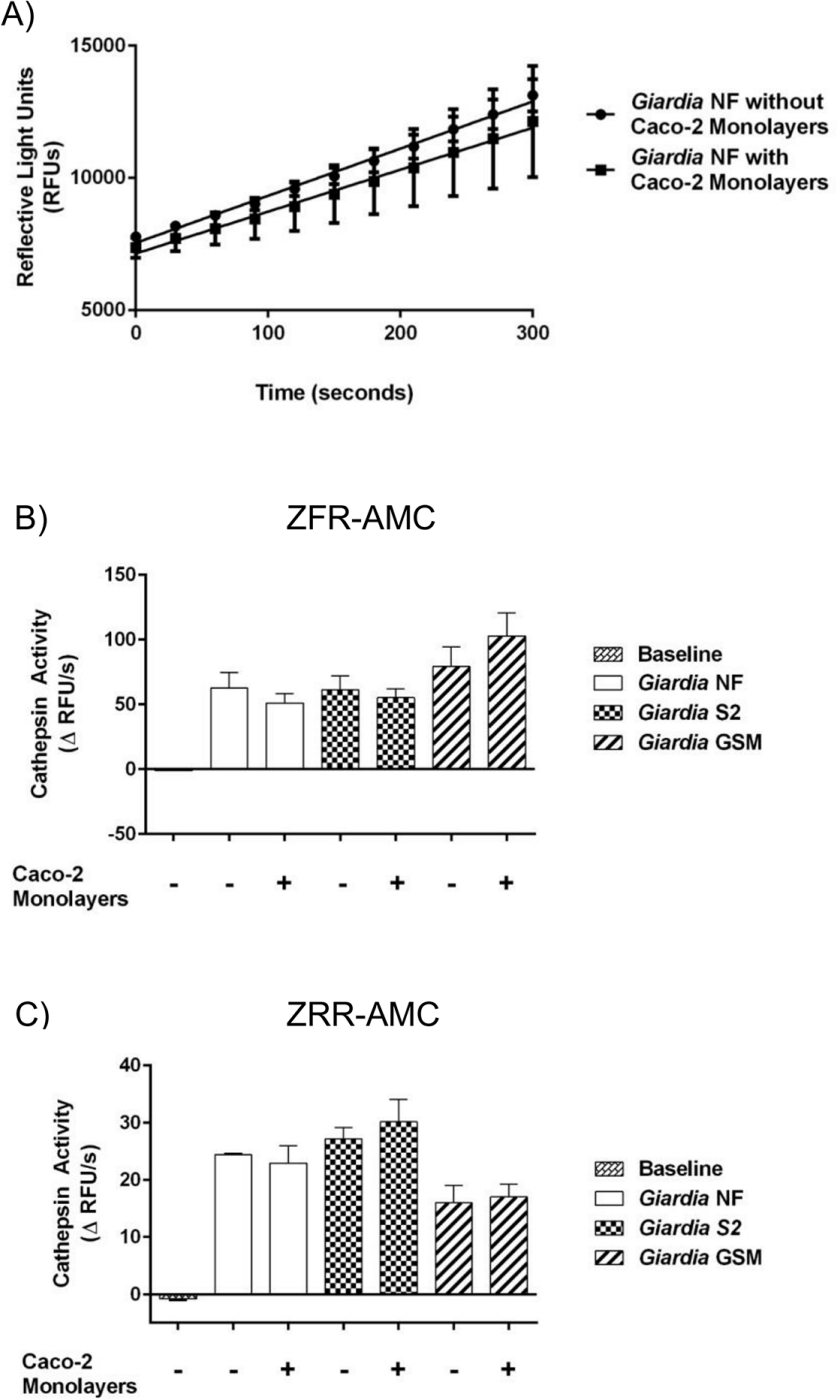
*Giardia duodenalis* trophozoite sonicates hydrolyze cathepsin cysteine protease substrates in an isolate-independent manner. This figure illustrates cathepsin activity within trophozoite sonicates. *G*. *duodenalis* trophozoites (isolates NF, S2, or GS/M) were incubated in the presence or absence of Caco-2 monolayers for 24 hours. Following this, *G*. *duodenalis* trophozoites were collected and sonicated to assess for intra-trophozoite cathepsin activity (A to C). The slope value was calculated from the RFU vs time for each experimental group. Line graphs are not shown for all to avoid redundancy, but a representative graph is shown for isolate NF. As a representative figure, *G*. *duodenalis* NF trophozoite sonicates were incubated with the catB/L substrate ZFR-AMC (200 μM: 5 min: 37°C: pH 7.2). Proteolytic activity is represented as the change in RFUs over time (A). *G*. *duodenalis* trophozoite sonicates (isolates NF, S2, or GS/M) or culture media alone (baseline) were incubated with the catB/L fluorogenic substrate ZFR-AMC (B) or the catB-specific fluorogenic substrate (C) (ZRR-AMC) (200 μM: 5 min: 37°C: pH 7.2). Proteolytic activity was calculated by determining the change or slope in RFUs over time. Data are mean+/-SEM, n = 3.

**Fig 4:**
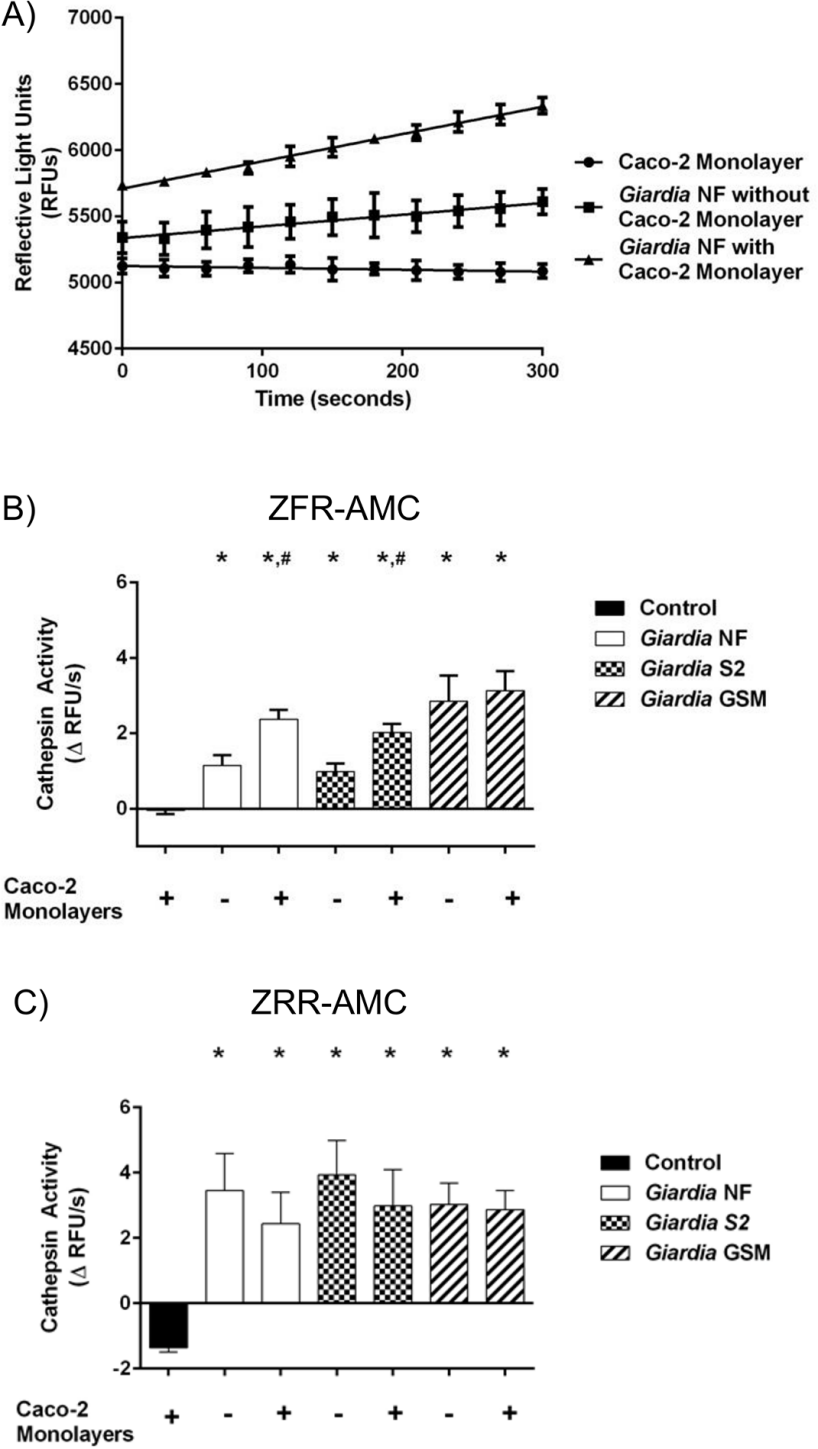
*Giardia duodenalis* trophozoites release catB and L cysteine proteases into cell supernatants. This figure illustrates cathepsin activity in parasite supernatants (released activity from trophozoites). *G*. *duodenalis* trophozoites (isolates NF, S2, or GS/M) were incubated in the presence or absence of Caco-2 monolayers for 24 hours. Supernatants were collected and analyzed for cathepsin cysteine protease activity (A to C). As a representative figure, supernatants collected from *G*. *duodenalis* NF incubations in the presence or absence of Caco-2 monolayers were incubated with the catB/L fluorgenic substrate ZFR-AMC (200 μM: 5 min: 37°C: pH 7.2). Proteolytic activity is represented as the change in RFUs over time (A). Supernatants were collected and incubated with the catB/L fluorogenic substrate ZFR-AMC (B) or the catB-specific fluorogenic substrate (C) (ZRR-AMC) (200 μM: 5 min: 37°C: pH 7.2). Proteolytic activity was calculated by determining the change or slope in RFUs over time.). *p<0.05 vs Control monolayers #p<0.05 vs corresponding isolate incubated without Caco-2 monolayers. Data are mean+/-SEM, n = 3.

**Fig 5:**
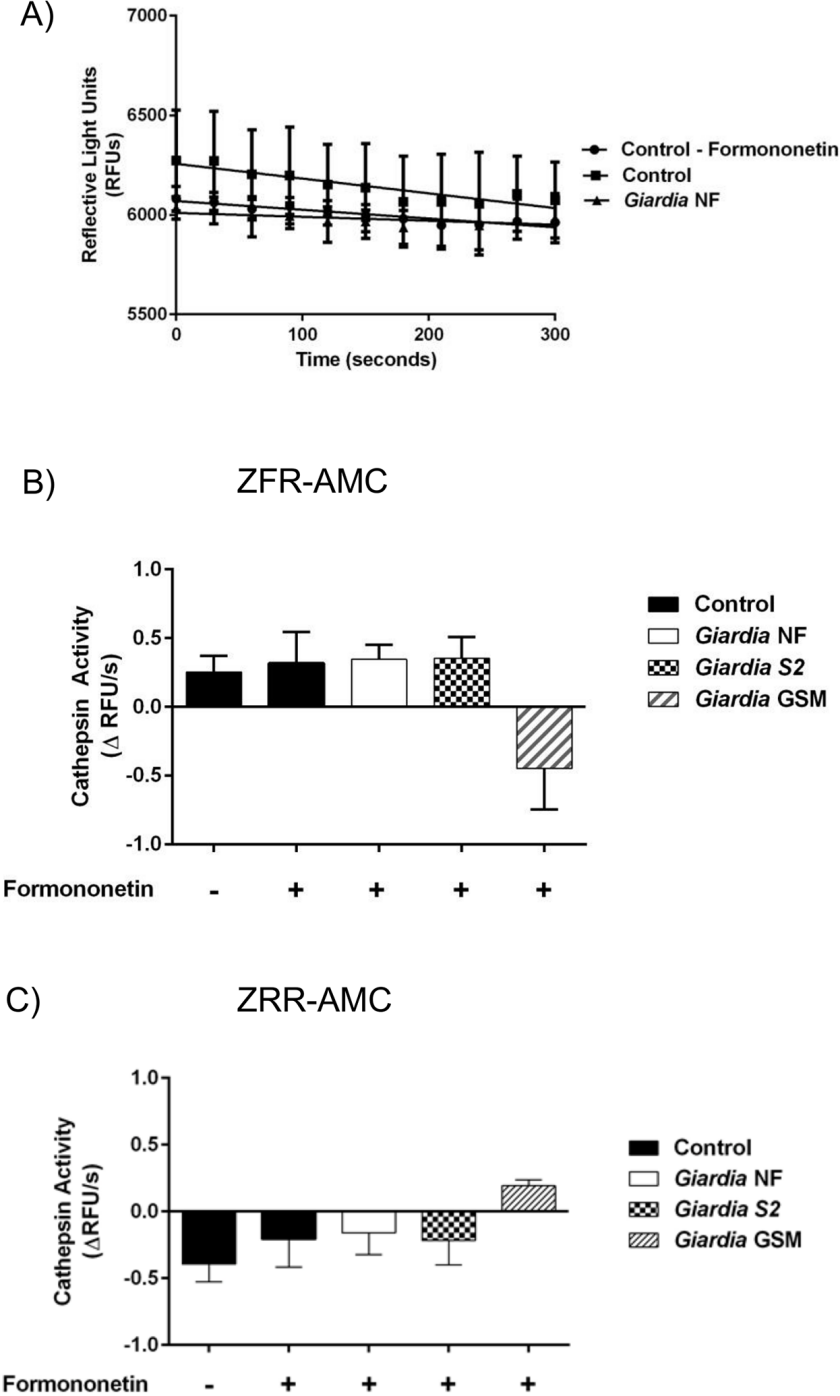
*Giardia duodenalis* trophozoites do not induce catB/L activity within Caco-2 monolayers. This figure illustrates cathepsin activity within Caco-2 cells post co-incubation with trophozoites. *G*. *duodenalis* trophozoites (isolates NF, S2, or GS/M) were co-incubated with Caco-2 monolayers for 24 hours. Caco-2 cell lysates were collected and analyzed for cathepsin cysteine protease activity (A to C). As a representative figure, Caco-2 lysates co-incubated with *G*. *duodenalis* NF trophozoites were incubated with the catB/L fluorogenic substrate ZFR-AMC (200 μM: 5 min: 37°C: pH 7.2). Proteolytic activity is represented as the change in RFUs over time (A). Caco-2 lysates were collected and incubated with the catB/L fluorogenic substrate ZFR-AMC (B) or the catB-specific fluorogenic substrate (C) (ZRR-AMC) (200 μM: 5 min: 37°C: pH 7.2). Proteolytic activity was calculated by determining the change or slope in RFUs over time. Data are mean+/-SEM, n = 3.

Previous research has demonstrated that *G*. *duodenalis* trophozoites contain, as well as release, cathepsin cysteine proteases into cell supernatants [28–31, 34]. However, the kinetics of cathepsin cysteine protease activity within multiple *G*. *duodenalis* trophozoites, IECs, or cell supernatants during co-incubation of parasites and IECs remain unknown. Therefore, cathepsin cysteine protease activity levels were assessed within parasites, IECs, and cell supernatants, following co-incubation. Cathepsin cysteine protease activity was determined by calculating the slope from the RFU versus time, as illustrated in Fig 3A. Figs 3B, 3C, 4B, 4C, 5B and 5C illustrate these calculated slopes and are shown as histograms. As demonstrated by an increase in the number of detectable RFUs over time, hydrolysis of the catB and L fluorogenic substrate ZFR-AMC occurred in *G*. *duodenalis* NF trophozoite sonicates alone (Fig 3A), or cell supernatants from co-incubation (Fig 4A) with Caco-2 monolayers; in contrast, hydrolysis was not observed inside the Caco-2 cells incubated in the presence or absence of *G*. *duodenalis* NF trophozoites (Fig 5A).

Slope values were determined when *G*. *duodenalis* trophozoites (NF, S2, or GS/M) were co-incubated in Caco-2 growth media in the presence or absence of Caco-2 monolayers. Hydrolysis of the catB/L substrate ZFR-AMC (Fig 3B) and catB-selective substrate ZRR-AMC (Fig 3C) was observed in sonicates from tested *G*. *duodenalis* isolates (NF, S2, or GS/M) and values were not statistically significant from each other (Fig 3B and 3C). A significant increase in ZFR-AMC (Fig 4B) and ZRR-AMC (Fig 4C) hydrolysis was detected in cell supernatants collected from *G*. *duodenalis* trophozoites incubated Caco-2 growth media in the presence or absence of Caco-2 monolayers, when compared against control groups. Interestingly, co-incubation of *G*. *duodenalis* NF or S2 trophozoites with Caco-2 monolayers significantly increased cell supernatant hydrolysis of ZFR-AMC, compared against the same isolate incubated in the absence of Caco-2 cells (Fig 4B). This trend was not observed when hydrolysis of ZRR-AMC was analyzed (Fig 4C). Hydrolysis of ZFR-AMC (Fig 5B) and ZRR-AMC (Fig 5C) was not significantly increased in Caco-2 monolayers incubated in the presence or absence of *G*. *duodenalis* trophozoites. Therefore, *G*. *duodenalis* trophozoites do not appear to increase catB/L activity inside Caco-2 monolayers. These results demonstrate that catB/L cysteine protease activities are active at similar levels within *G*. *duodenalis* trophozoites, and that parasites release cysteine proteases into cell supernatants. However, this activity was further increased in two Assemblage A isolates (NF and S2) following their exposure to Caco-2 monolayers, while this further increase could not be detected when cells were co-incubated with the Assemblage B GS/M.

**Fig 6:**
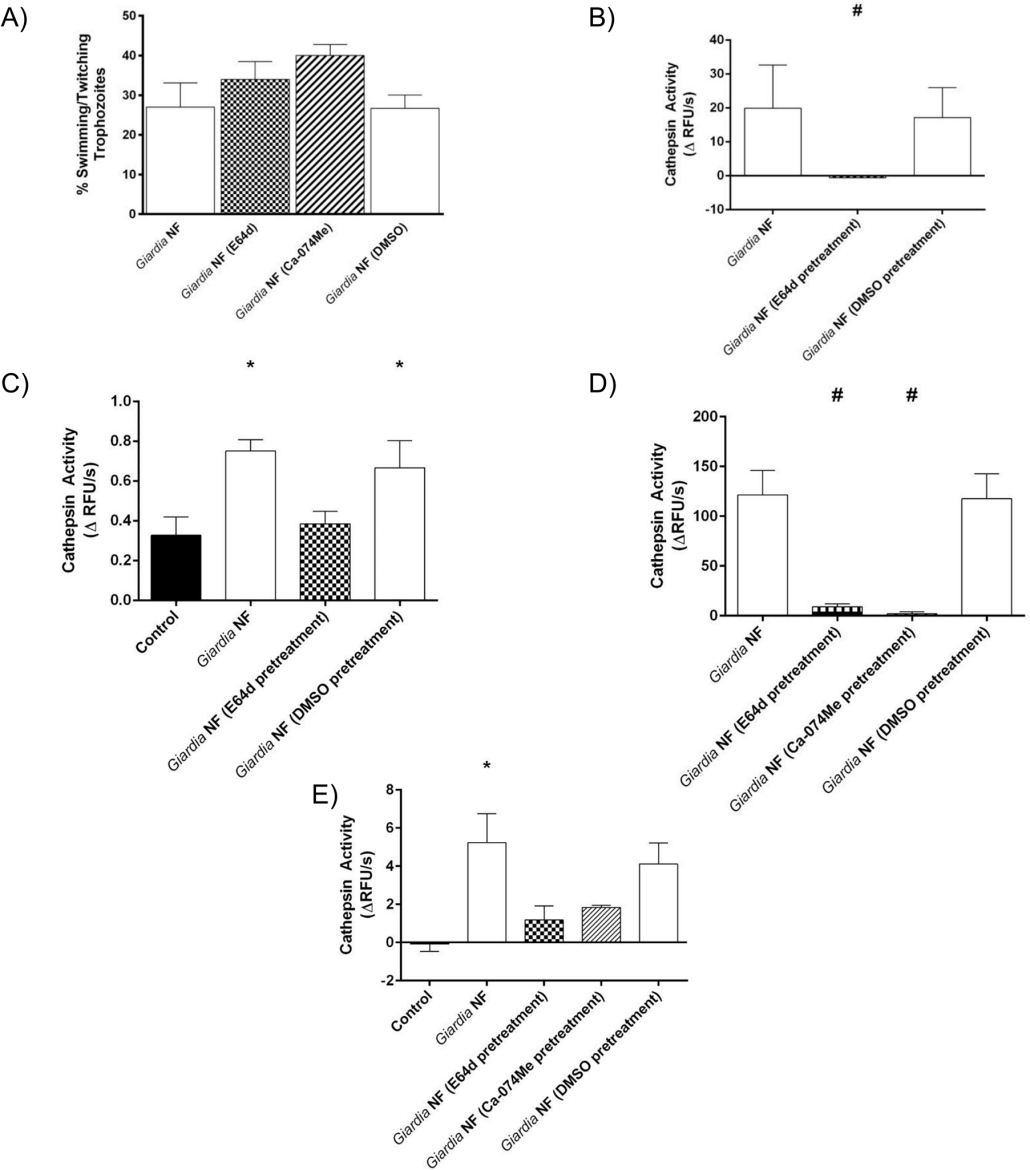
Pre-treatment of *Giardia duodenalis* trophozoites with E-64d or Ca-074Me inhibits cathepsin cysteine protease activity. *G*. *duodenalis* NF trophozoites were treated with E-64d (10uM), Ca074Me (10uM), or vehicle control (DMSO) for 3 hours and then co-incubated with Caco-2 monolayers for 2 (B and C) or 24 (D and E) hours. *G*. *duodenalis* trophozoites were collected and sonicated to assess for intra-trophozoite cathepsin cysteine protease activity. Supernatants were collected and assessed for the viability of *G*. *duodenalis* trophozoites by examining the ratio of motile: non-motile trophozoites (A). *G*. *duodenalis* sonicates were incubated with catB/L fluorogenic substrate ZFR-AMC (B and D) or the catB fluorogenic substrate ZRR-AMC (C and E) (200 μM: 5 min: 37°C: pH 7.2). Proteolytic activity was calculated by determining the change in RFUs over time. *p<0.05 vs Control cells; #p<0.05 vs G. *duodenalis* NF trophozoites. Data are mean +/- SEM, n = 3.

*G*. *duodenalis* NF trophozoites were used for the rest of the study to assess whether and how parasite catB/L proteases may affect host IECs. Initial experiments sought to determine whether catB/L activity could be inhibited within *G*. *duodenalis* trophozoites via the broad-spectrum clan CA cysteine protease inhibitor E64d (10 μM) or the catB-specific inhibitor Ca-074Me (10 μM). Parasite viability was not affected by 3-hour treatment with E-64d or Ca-074Me; compared against control *G*. *duodenalis* trophozoites, no significant difference in the proportion of motile to non-motile trophozoites in groups treated with E64d, Ca-074Me, or vehicle control (DMSO) was observed (Fig 6A). Next, Caco-2 monolayers were co-incubated with *G*. *duodenalis* NF trophozoites pretreated with E64d for 2 or 24 hours; this treatment significantly reduced the hydrolysis of ZFR-AMC within *G*. *duodenalis* sonicates (Fig 6B) and cell supernatants (Fig 6C), following their 2-hour co-incubation with Caco-2 monolayers. Similarly, hydrolysis of ZFR-AMC was significantly reduced within *G duodenalis* sonicates (Fig 6D) and cell supernatants (Fig 6E) when parasites were pre-treated with E-64d or Ca-074Me for 3-hours and subsequently co-incubated with Caco-2 monolayers for 24 hours. Collectively, these results demonstrate that *G*. *duodenalis* catB and L proteases are sensitive to inhibition using commercial protease inhibitors.

### *Giardia duodenalis* cathepsin cysteine proteases promote villin breakdown in a contact-dependent manner

Recent research has focused on the ability of *G*. *duodenalis* to disrupt tight junctional proteins [37, 38, 40, 69]. Less research has focused on this parasite’s ability to affect intestinal epithelial cytoskeletal proteins. *G*. *duodenalis* disrupts cytoskeletal actin filaments in a myosin light chain kinase (MLCK)-dependent manner [39]. Moreover, host CD4+ and CD8+ T-lymphocytes are responsible for villin cleavage during late-stage *G*. *duodenalis* infection *in vivo* [51]. It is not known whether *G*. *duodenalis* parasite products are capable of directly targeting intestinal epithelial cytoskeletal proteins such as villin. As previous findings have shown that *Entamoeba histolytica* proteolytically degrades villin using cysteine proteases [70], we hypothesized that *G*. *duodenalis* cathepsin-like cysteine proteases was able to degrade and disrupt villin.

**Fig 7:**
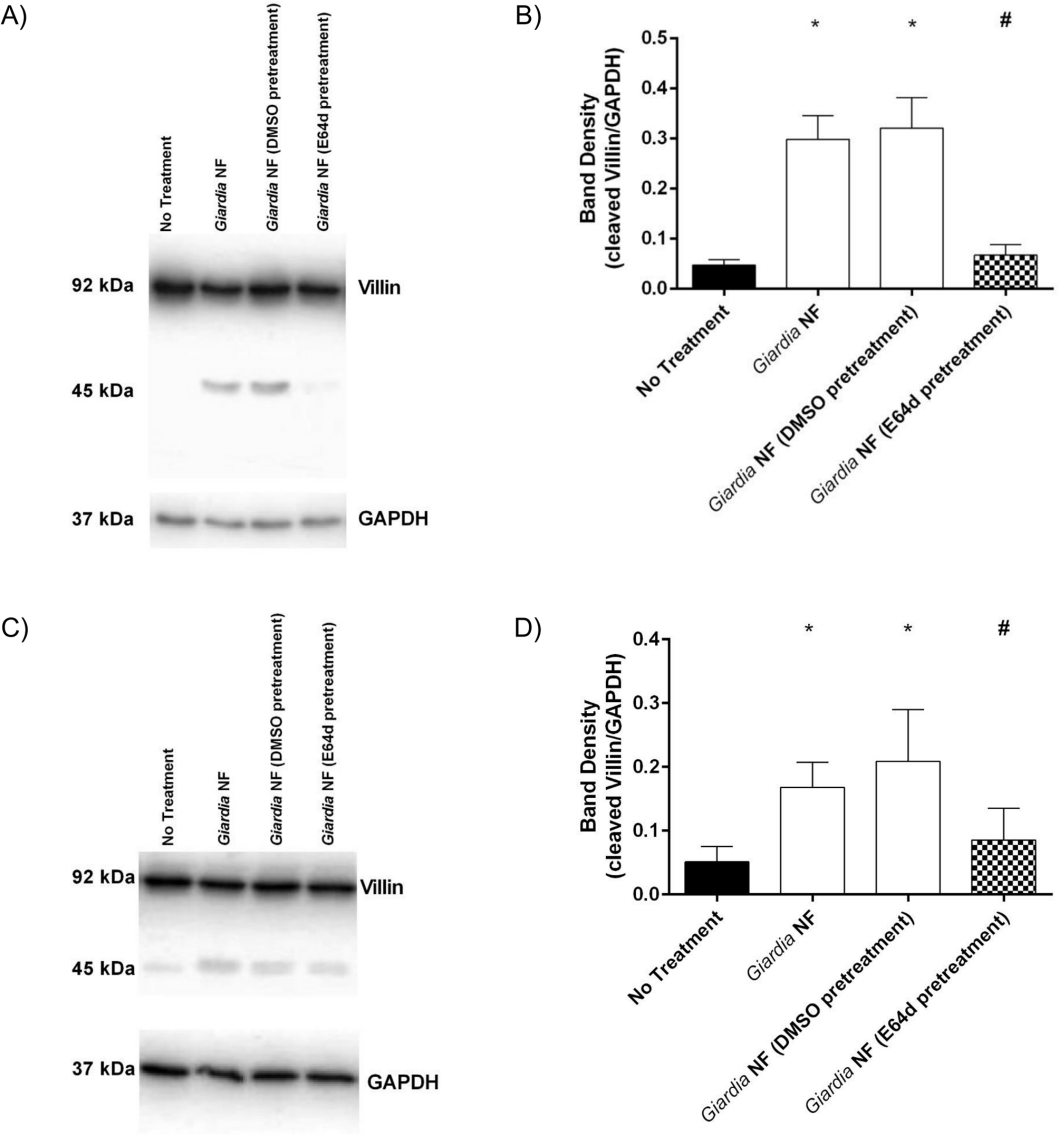
*Giardia duodenalis* NF trophozoite proteases that are sensitive to inhibition with E-64d promote villin cleavage in Caco-2 monolayers. *G*. *duodenalis* NF isolate trophozoites were pre-treated with E64d (10uM) or vehicle control (DMSO) prior to co-incubation with Caco-2 monolayers for 2 (A and B) or 24 (C and D) hours. Caco-2 lysates were collected and processed for Western blotting to examine for villin protein at 2 (A) and 24 (C) hours. Western blots are representative of three independent experiments performed in triplicate. Densitometry was performed to compare protein levels of cleaved villin vs loading control GAPDH for the 2 (B) or 24 (D) hour co-incubation. *p<0.05 vs No Treatment. Data are mean +/- SEM, n = 3.

**Fig 8:**
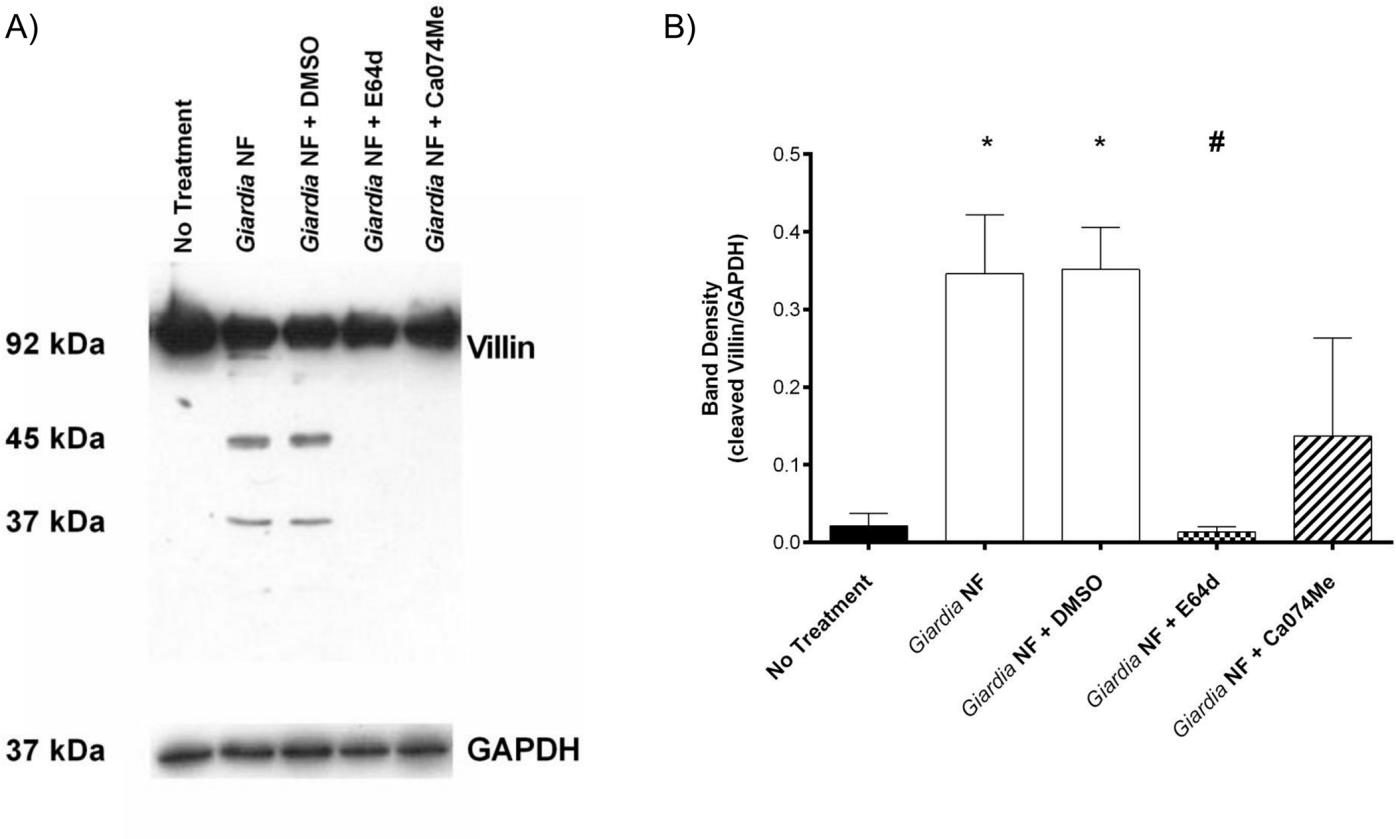
Co-incubation of *Giardia duodenalis* NF trophozoite sonicates and Caco-2 lysates results in villin cleavage and is prevented by E64d. *G*. *duodenalis* NF isolate trophozoite sonicates and Caco-2 monolayers were co-incubated for 2 hours in the presence of E-64d (10uM), Ca-74Me (10uM), or vehicle control (DMSO). Samples were processed Western blotting to examine for villin protein (A). Densitometry was performed to compare protein levels of cleaved villin vs loading control GAPDH (B). *p<0.05 vs No Treatment. Data are mean +/- SEM, n = 3.

**Fig 9:**
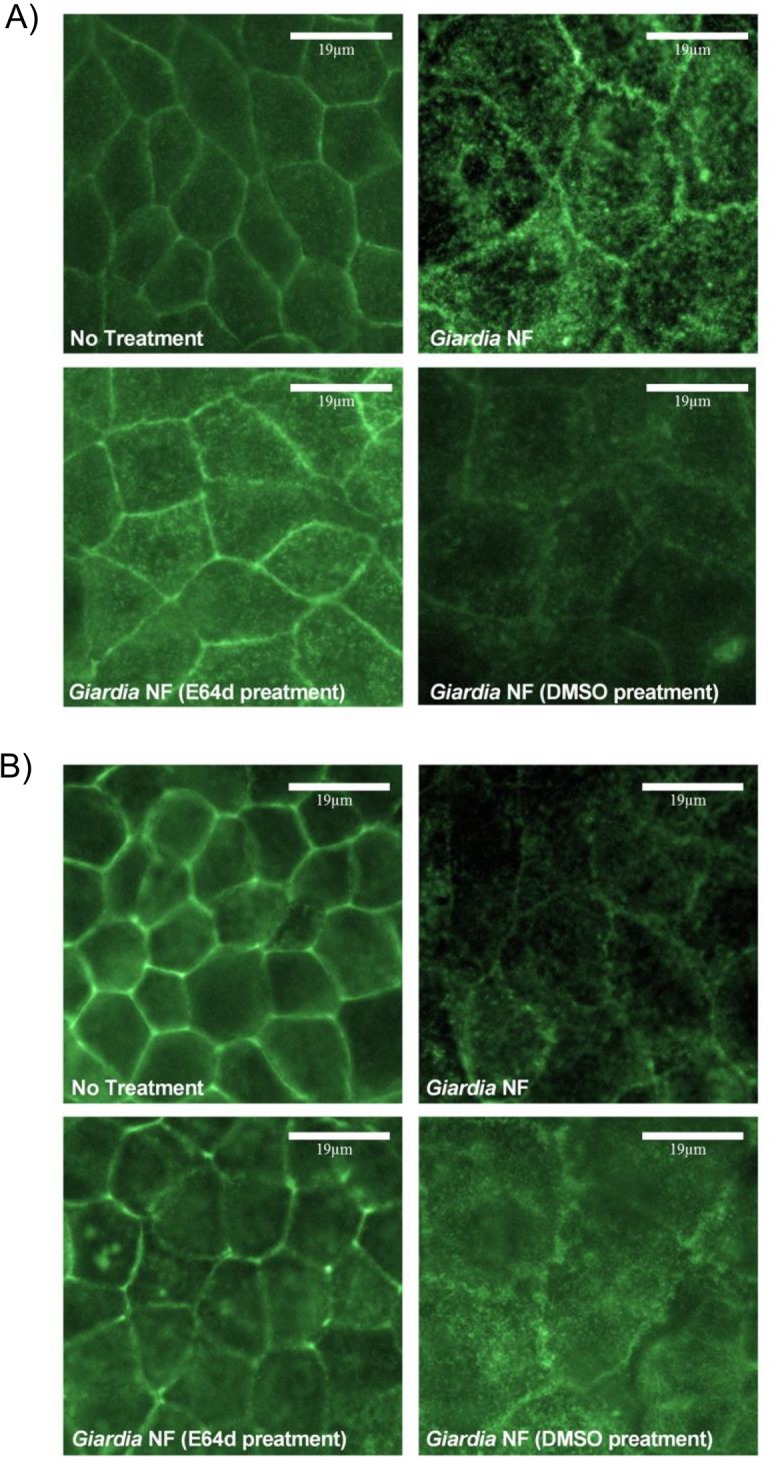
*Giardia duodenalis* trophozoites produce proteases that are sensitive to inhibition with E-64 that induce disruption of villin within Caco-2 monolayers. *G*. *duodenalis* NF isolate trophozoites were pre-treated with E64d (10uM) or vehicle control (DMSO) prior to co-incubation with Caco-2 monolayers for 2 (A) or 24 (B) hours. Caco-2 monolayers were processed for immunofluorescence to examine for villin expression at 2 (A) or 24 (B) hours at 400X magnification. Micrographs are representative of three independent experiments performed in duplicate. n = 3.

Co-incubation of *G*. *duodenalis* NF trophozoites with Caco-2 monolayers for 2 hours yielded a ~ 45kDa cleavage product as determined by Western blotting (Fig 7A and 7B). Full-length villin (90kDa) was not altered, as determined via densitometry (Fig 7B). Similar results were observed when parasites and Caco-2 monolayers were co-incubated for 24 hours as illustrated from Western blotting and densitometric analyses (Fig 7C and 7D). Densitometry also demonstrated that E-64d pre-treatment of *G*. *duodenalis* NF trophozoites prior to co-incubation with monolayers resulted in decreased detection of the villin cleavage product at both 2 (Fig 7B) and 24 (Fig 7D) hours. Follow-up experiments were performed to determine whether co-incubation of parasite sonicates with Caco-2 cellular lysates for 2 hours also resulted in villin cleavage. As demonstrated via Western blotting and densitometry, co-incubation of *G*. *duodenalis* trophozoite sonicates with Caco-2 cellular lysates resulted in increased detection of villin cleavage fragments (Fig 8A and 8B). Importantly, this was significantly reversed when experiments were performed in the presence of E-64d (Fig 8A and 8B). These results suggest that *G*. *duodenalis* parasite products are capable of cleaving villin within intestinal epithelial cellular lysates. Similarly, immunofluorescent staining indicated that co-incubation of *G*. *duodenalis* trophozoites with Caco-2 monolayers resulted in redistribution of villin protein at 2 (Fig 9A) and 24 (Fig 9B) hours. Importantly, pre-treatment of *G*. *duodenalis* trophozoites with E-64d, at least partially, prevented redistribution of villin within IECs at 2 and 24 hours (Fig 9A and 9B).

**Fig 10:**
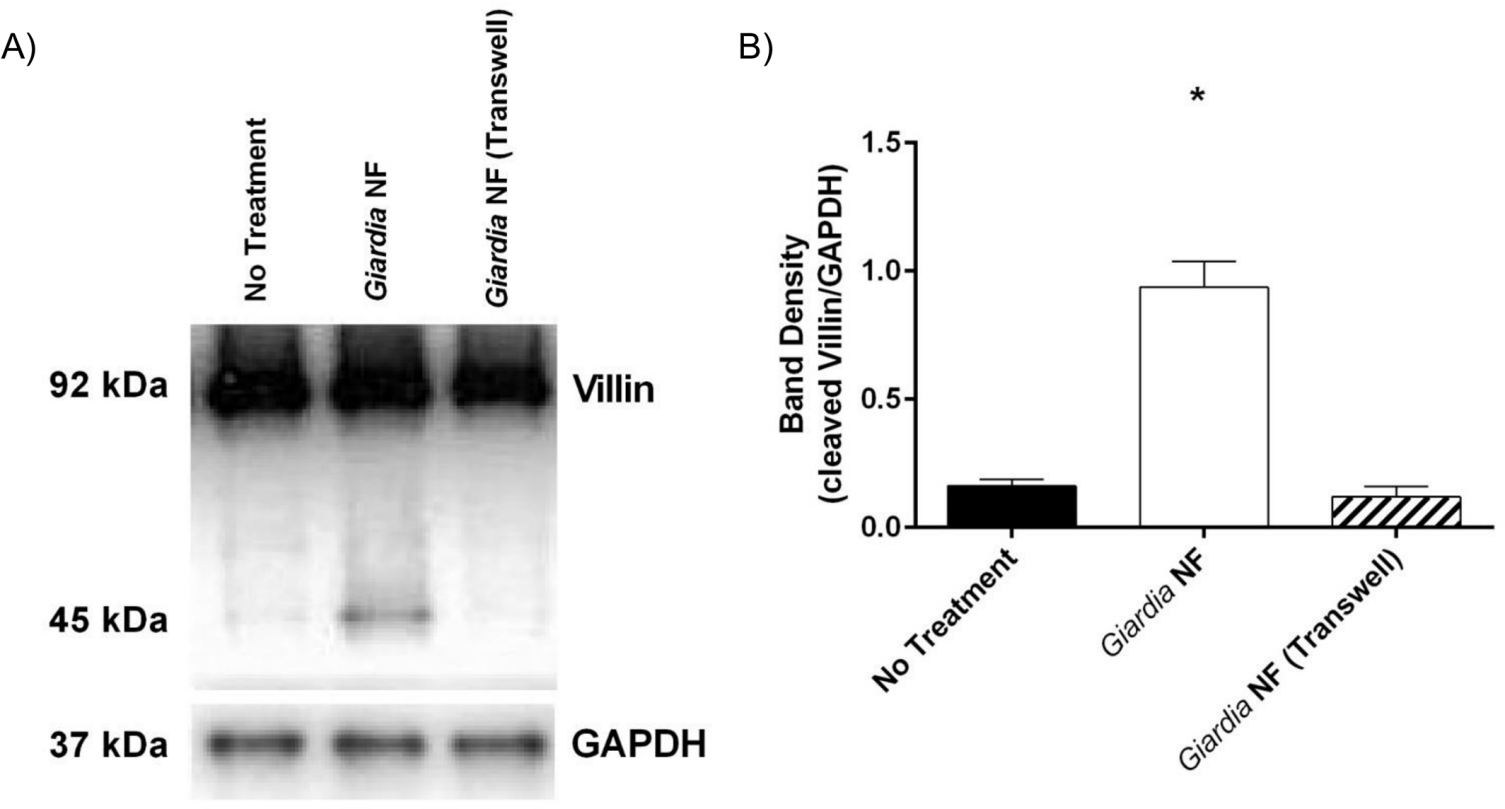
*Giardia duodenalis* induces villin cleavage in Caco-2 monolayers in a contact-dependent manner. *G*. *duodenalis* NF isolate trophozoites were co-incubated directly contacting Caco-2 monolayers or separated from Caco-2 monolayers via 0.4um Transwells for 24 hours. Caco-2 lysates were collected and processed for Western blotting to examine for villin protein (A). Western blots are representative of three independent experiments performed in triplicate. Densitometry was performed to compare protein levels of cleaved villin vs loading control GAPDH (B). *p<0.05 vs No Treatment. Data are mean +/- SEM, n = 3.

To determine whether secreted products were sufficient to cause a villin break down, parasites and Caco-2 monolayers were separated by 0.4μm transwells and co-incubated for 24 hours; interestingly, Western blotting and subsequent densitometry indicated that villin breakdown was not seen in these groups (Fig 10A and 10B). Together, these results demonstrate that *G*. *duodenalis* clan CA cysteine proteases are involved in the cleavage of cytoskeletal villin in Caco-2 monolayers. Moreover, these data suggest that a parasite surface clan CA cysteine protease contributes to villin cleavage and redistribution within Caco-2 monolayers.

### Giardia duodenalis clan CA cysteine proteases do not contribute to ZO-1 breakdown

**Fig 11:**
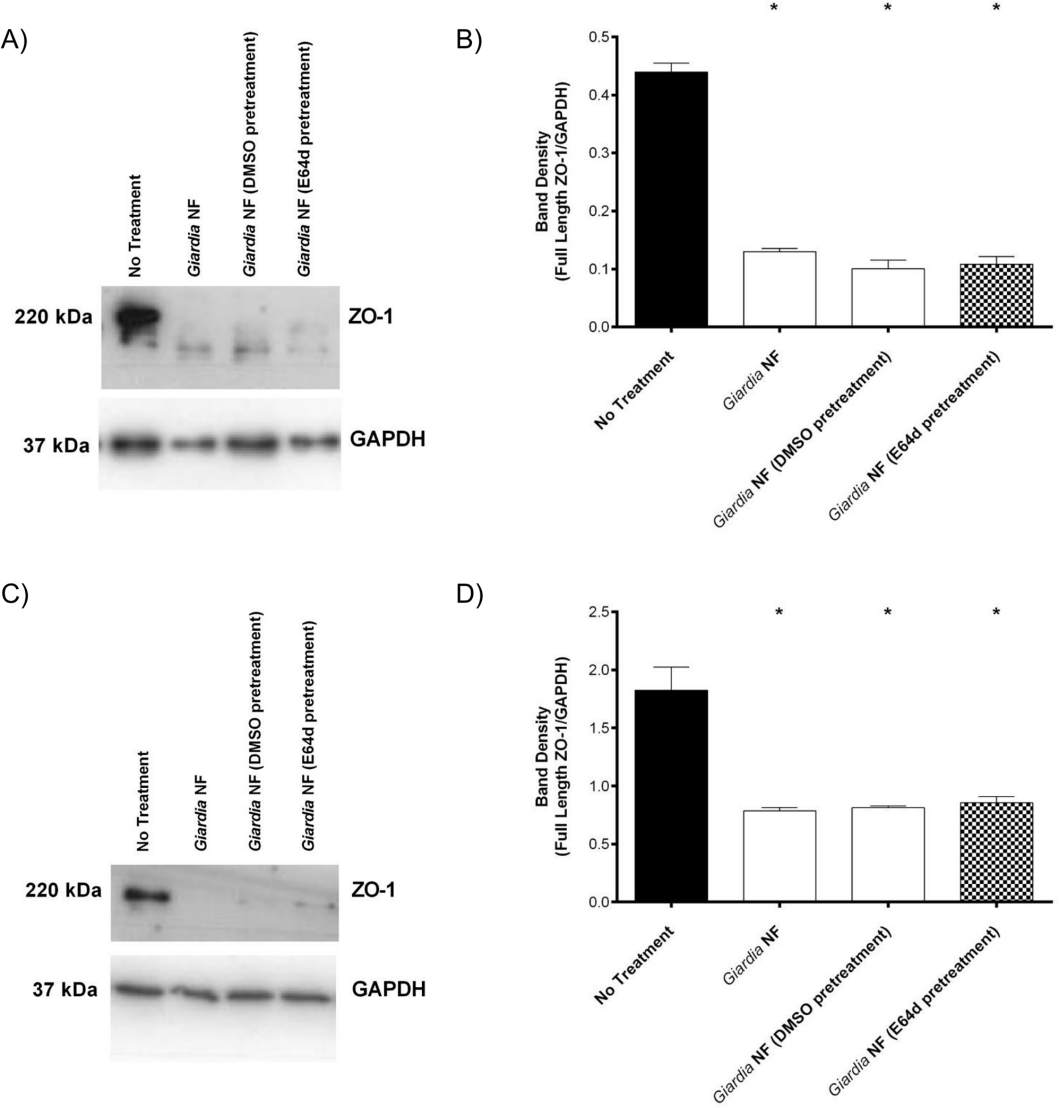
Pre-treatment with E-64d does not inhibit *Giardia duodenalis* trophozoites from-inducing ZO-1 degradation in Caco-2 monolayers. *G*. *duodenalis* NF isolate trophozoites were pre-treated with E64d (10uM) or vehicle control (DMSO) prior to co-incubation with Caco-2 monolayers for 2 (A and B) or 24 (C and D) hours. Caco-2 cell lysates were collected and processed for Western blotting to examine for ZO-1 protein at 2 (A) and 24 (C) hours. Western blots are representative of three independent experiments performed in triplicate. Densitometry was performed to compare full length ZO-1 levels vs loading control GAPDH after 2 (B) or 24 (D) hours. *p<0.05 vs Control cells. Data are mean +/- SEM, n = 3.

Previous research has demonstrated that *G*. *duodenalis* trophozoites promote the breakdown and redistribution of the tight junction-associated protein zonula occludens 1 (ZO-1) [37–39, 69]. Therefore, we decided to assess whether *G*. *duodenalis* clan CA cysteine proteases affect ZO-1 in a manner similar to villin. As determined via Western blotting and corresponding densitometry, co-incubation of *G*. *duodenalis* trophozoites with Caco-2 monolayers resulted in loss of full-length ZO-1 protein at 2 (Fig 11A and 11B) and 24 (Fig 11C and 11D) hours. However, pre-treatment of *G*. *duodenalis* trophozoites with E-64d, and subsequent incubation with Caco-2 monolayers, did not affect degradation of ZO-1 at 2 (Fig 11A and 11B) or 24 hours (Fig 11C and 11D). These observations suggest that degradation and redistribution of villin and ZO-1 may occur via separate mechanisms.

### *Giardia duodenalis*-induced villin breakdown is partially dependent on myosin light chain kinase

**Fig 12:**
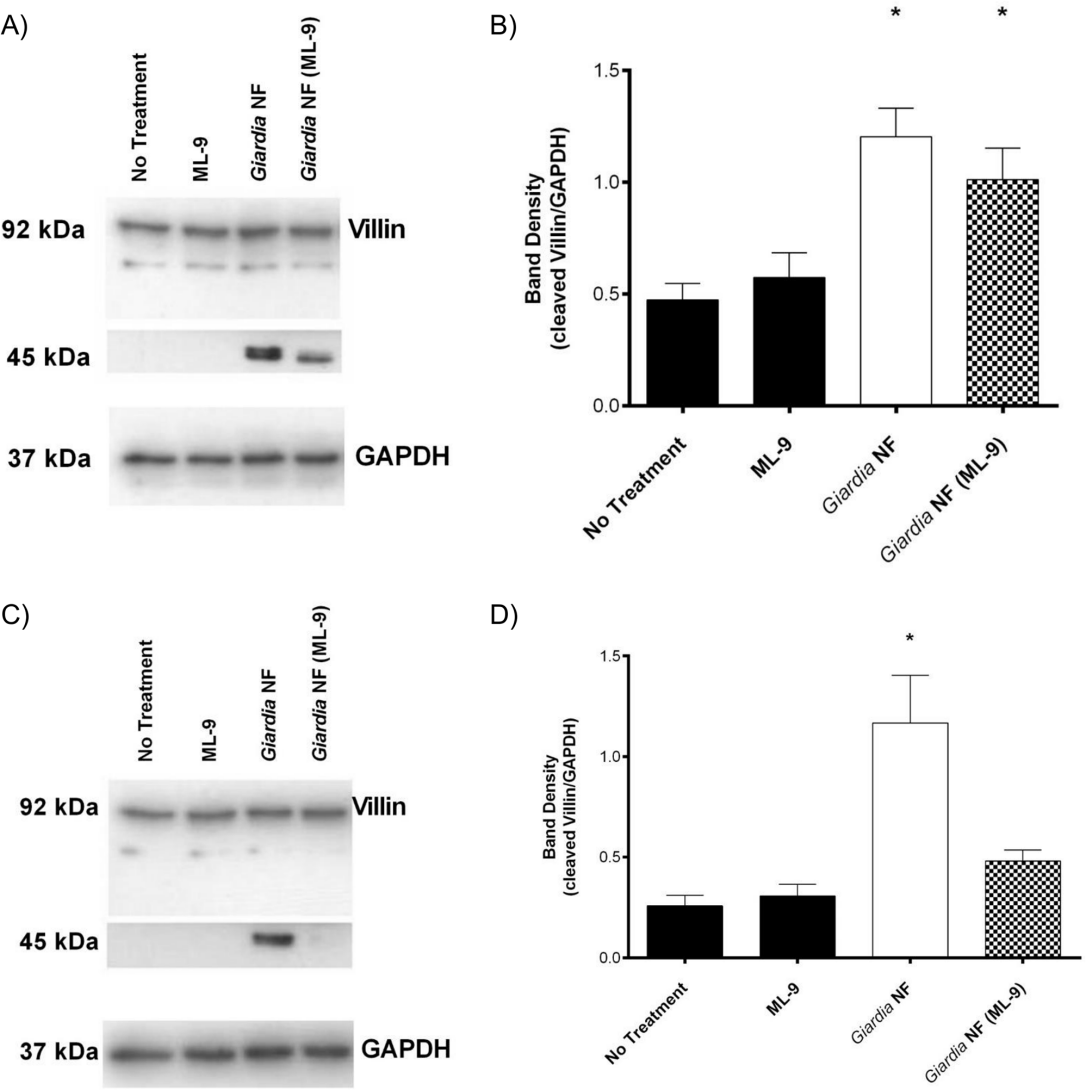
*Giardia duodenalis* trophozoites induce MLCK-dependent cleavage of villin in Caco-2 monolayers in a time-dependent manner. *G*. *duodenalis* NF isolate trophozoites were co-incubated with Caco-2 monolayers for 2 (A and B) or 24 (C and D) hours in the presence or absence of the MLCK inhibitor ML-9. Caco-2 cell lysates were processed for Western blotting to examine for villin protein at 2 (A) and 24 (C) hours. Western blots are representative of three independent experiments performed in triplicate. Densitometry was performed to compare protein levels of cleaved villin vs loading control GAPDH after a 2 (B) or 24 (D) hour co-incubation. *p<0.05 vs No Treatment. Data are mean +/- SEM, n = 3.

**Fig 13:**
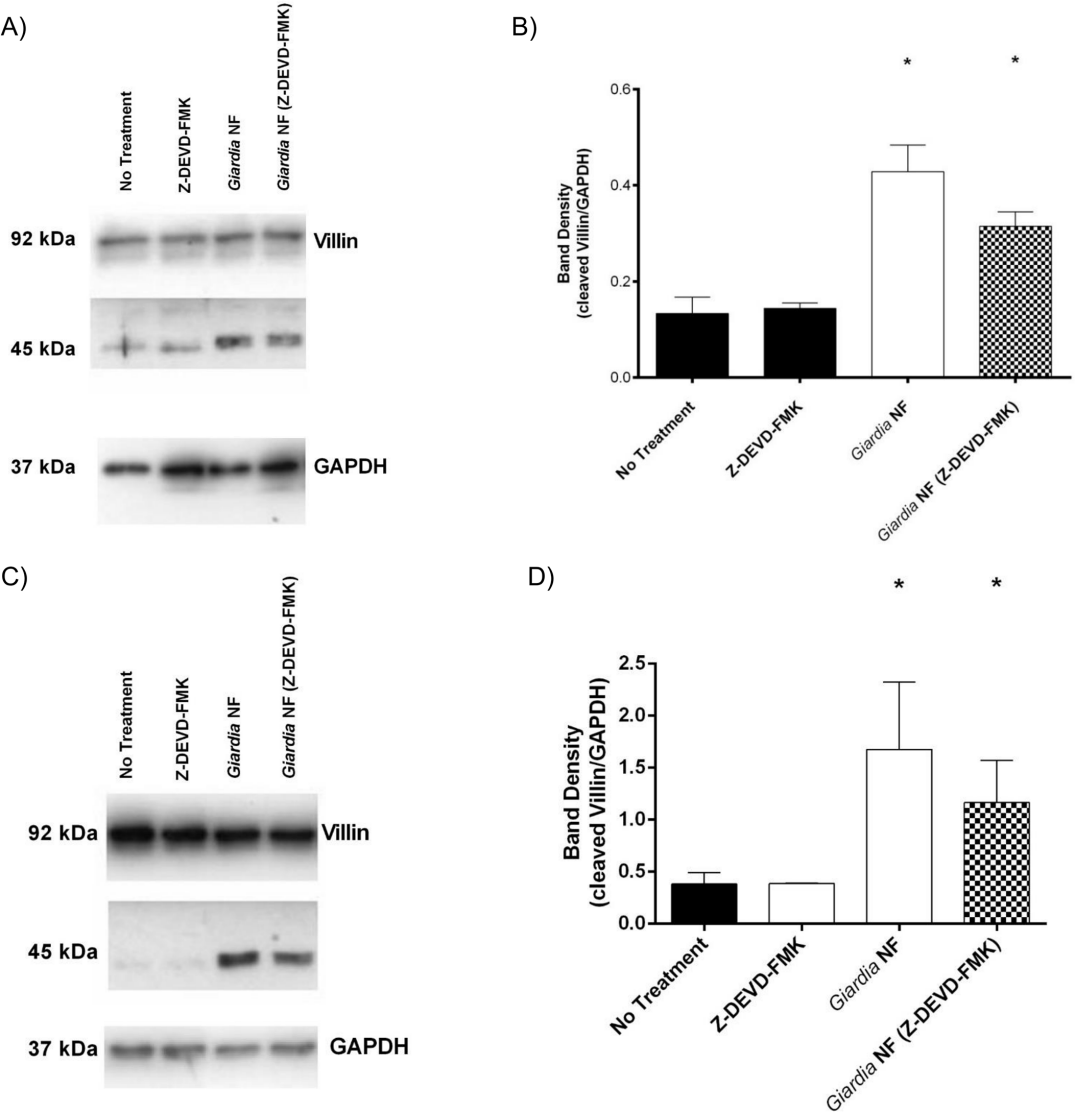
*Giardia duodenalis* trophozoites promote villin cleavage in Caco-2 monolayers independent of caspase-3 activity. *G*. *duodenalis* NF isolate trophozoites were co-incubated with Caco-2 monolayers for 2 (A and B) or 24 (C and D) hours in the presence or absence of Z-DEVD-FMK, a selective caspase-3 inhibitor. Caco-2 cell lysates were collected and processed for Western blotting to examine for villin protein at 2 (A) and 24 (C) hours. Western blots are representative of three independent experiments performed in triplicate. Densitometry was performed to compare protein levels of cleaved villin vs loading control GAPDH at 2 (B) and 24 (D) hours. *p<0.05 vs No Treatment. Data are mean +/- SEM, n = 3.

As *Giardia* is known to disrupt tight junctional proteins by activating MLCK and caspase-3 [38, 39]), we decided to assess whether *G*. *duodenalis* clan CA cysteine proteases induce villin disruption via activation of MLCK or caspase-3. Therefore, experiments were performed whereby Caco-2 monolayers were pre-treated with the MLCK-selective inhibitor ML-9 or the caspase-3-specific inhibitor Z-DEVD-FMK prior to co-incubation with *G*. *duodenalis* trophozoites for 2 or 24 hours. Pre-treatment of Caco-2 monolayers with ML-9 prior to co-incubation with *G*. *duodenalis* trophozoites for 2 hours did not affect villin cleavage, as determined via Western blotting analysis (Fig 12A and 12B). Interestingly, villin degradation was significantly reduced in Caco-2 monolayers pre-treated with ML-9 prior to co-incubation with *G*. *duodenalis* trophozoites for 24 hours (Fig 12C and 12D). No significant difference in villin cleavage was observed when Caco-2 monolayers were pre-treated with Z-DEVD-FMK and co-incubated with *G*. *duodenalis* trophozoites for 2 (Fig 13A and 13B) or 24 hours (Fig 13C and 13D). Collectively, these results demonstrate that *G*. *duodenalis*-mediated villin degradation and redistribution is, at least partially, dependent on MLCK activation.

## Discussion

Results from this study reveal a previously unrecognized role for parasite surface clan CA cysteine proteases in the pathophysiology of *G*. *duodenalis* infections, and implicate them as potential virulence factors. CatB/L activity was observed within *G*. *duodenalis* trophozoites and in parasite supernatants following exposure to host IECs. This activity was inhibited when parasites were pre-treated with the broad-spectrum clan CA, cysteine protease inhibitor E-64d [52] or the catB-specific inhibitor Ca-074Me [55]. Co-incubation of the parasite with IEC’s increased the production of catB and L activity for Assemblage A, but not for the Assemblage B isolate. However, no cathepsin activity was observed within the Caco-2 cells following exposure to *G*. *duodenalis* trophozoites from any isolate, ruling out any translocation of the cysteine proteases into the cells from the supernatant. *G*. *duodenalis* trophozoites degraded and redistributed the intestinal epithelial cytoskeletal protein villin; this required direct contact between the parasite and IEC, and was prevented when parasites were pre-treated with E-64d prior to incubation with intestinal monolayers. In addition, this study elucidated that degradation and redistribution of the tight junction protein ZO-1 was not dependent on factors sensitive to inhibition with E-64d, thereby suggesting that *G*. *duodenalis* may induce multiple pathophysiological responses within host IECs, some mediated by parasite clan CA cysteine proteases, some not. Finally, this study demonstrates that degradation and disruption of villin is partially dependent upon MLCK activation. At early time points, MLCK inhibition in intestinal monolayers exposed to *G*. *duodenalis* trophozoites did not prevent villin degradation; however, MLCK inhibition prevented *G*. *duodenalis*-induced villin degradation at later time points. Collectively, these results suggest that *G*. *duodenalis* surface clan CA cysteine proteases induce villin degradation and redistribution within IECs and that this effect, at least in part, is mediated by MLCK activation within IECs.

To elucidate the role of parasite cathepsin cysteine proteases in the pathophysiology of *G*. *duodenalis* infections, we used a well-established *in vitro* cell culture model [18, 19, 34, 57, 71, 72]. Furthermore, our experiments used inhibitors, which have previously been shown to successfully attenuate catB and L cysteine proteases in mammalian and protozoan parasites [24, 52, 55, 73], including *G*. *duodenalis* [34, 67]. Results from this study corroborate previous observations that *G*. *duodenalis* trophozoites promote the disruption of intestinal epithelial barrier proteins in vitro [37–40, 44, 69], and *in vivo* [46], and disrupt cytoskeletal protein villin [51]. These studies implicated host lymphocytes in the disruption of the intestinal epithelial cytoskeleton. In contrast, our study demonstrates that parasite clan CA cysteine proteases can directly disrupt intestinal epithelial villin.

Within the gastrointestinal tract, the cytoskeletal protein villin is exclusively expressed by IECs, and its primary function is to maintain microvillous brush border integrity during periods of stress via its ability to promote the assembly and disassembly of the actin cytoskeleton (reviewed in [49]). Pathogen-mediated actin cytoskeleton disruptions can aid in the establishment of infection [74–78]. Interestingly, inhibition of contact between parasites and IECs via chemical disruption of *G*. *duodenalis* lipid rafts prevents actin cytoskeletal remodelling [71]. Consistent with these observations, results from the present study demonstrate that *G*. *duodenalis* trophozoites were unable to induce villin degradation and redistribution within IECs when the two were separated by transwells. Collectively, these results suggest that intestinal epithelial actin cytoskeletal remodelling during giardiasis is dependent at least in part on contact between *G*. *duodenalis* trophozoites and host IECs. It remains to be elucidated whether actin cytoskeletal remodelling via villin activation is necessary for establishment of *G*. *duodenalis* infection.

Disruption of villin and the related protein ezrin occurs during the late stages of *G*. *duodenalis* infection *in vivo*, and is dependent on CD4+ and CD8+ lymphocytes [51]. Data from the present study indicate that early disruption of villin within IECs is dependent on clan CA cysteine proteases. Together, these results suggest that *G*. *duodenalis* uses more than one mechanism to disrupt villin and the intestinal epithelial brush border. A variety of invasive gastrointestinal pathogens are reliant on the intestinal epithelial expression of villin and its ability to remodel the actin cytoskeleton to facilitate entry and dissemination through host tissues [79–81]. Furthermore, although *Salmonella* sp. and enteropathogenic *Escherichia coli* induce villin redistribution, this occurs in the absence of villin proteolysis [82, 83]. Therefore, *G*. *duodenalis*-mediated disruption of villin may significantly affect the ability of other gastrointestinal pathogens to colonize and cause disease. Moreover, *G*. *duodenalis* infections have been reported to occur simultaneously with other pro-inflammatory gastrointestinal pathogens [84–87]. A recent report examining giardiasis in Tanzanian children found that individuals infected with *G*. *duodenalis* had reduced incidence of diarrhoeal disease and fever, and lower serum inflammatory scores when compared to individuals not infected with *G*. *duodenalis* [88]. More research is warranted to assess whether *G*. *duodenalis* infections may prevent other pathogens from establishing and/or inducing disease within their host.

Several parasites contain and produce cathepsin cysteine proteases (reviewed in [22, 23]). The *G*. *duodenalis* genome contains genes for multiple cathepsin B and L cysteine proteases, but their functions have only begun to be described [26]. Results illustrated herein indicate that *G*. *duodenalis* cathepsin cysteine proteases are involved in the degradation and redistribution of the intestinal epithelial cytoskeletal protein villin, in keeping with the observation that *Entamoeba histolytica* cysteine proteases promote villin breakdown [70]. The role of villin breakdown in the establishment of infection by enteric parasites requires further investigation. Prior to this study, *G*. *duodenalis* cathepsin cysteine proteases had been known to play a role in trophozoite encystation and excystation [33], and degrading the potent neutrophil chemoattractant interleukin-8 (CXCL8) [34]. Importantly, *G*. *duodenalis*-mediated attenuation of CXCL8 did not require direct contact between parasites and host IEC monolayers [34]. These results suggest *G*. *duodenalis* trophozoites possess multiple types of cathepsin cysteine proteases, and each may have a different role in disease pathogenesis. Indeed, single-celled parasites can possess both surface-associated and secreted cathepsin cysteine proteases which may have unique roles in pathogenesis [89–92].

In conclusion, our data reveal a novel role for *G*. *duodenalis* cathepsin cysteine proteases in the degradation and redistribution of the intestinal epithelial cytoskeletal protein villin. These results support previous observations that disruption of the intestinal epithelial cytoskeleton requires intimate contact between parasites and intestinal epithelial cells. The present findings also suggest that *Giardia* cysteine proteases disrupt villin at least in part by engaging epithelial MLCK. Our findings are the first to indicate that *G*. *duodenalis* cathepsin cysteine proteases represent potential parasite virulence factors capable of inducing pathophysiological responses within host intestinal epithelial cells.

## References

[1] Ankarklev J, Jerlstrom-Hultqvist J, Ringqvist E, Troell K, Svard SG. Behind the smile: cell biology and disease mechanisms of Giardia species. Nature reviews Microbiology. 2010;8(6):413–22. 10.1038/nrmicro2317 . 20400969

[2] Yoder JS, Harral C, Beach MJ, Centers for Disease C, Prevention. Giardiasis surveillance—United States, 2006–2008. Morbidity and mortality weekly report Surveillance summaries. 2010;59(6):15–25. . 20535095

[3] Olson ME, Guselle NJ, O'Handley RM, Swift ML, McAllister TA, Jelinski MD, et al Giardia and Cryptosporidium in dairy calves in British Columbia. The Canadian veterinary journal La revue veterinaire canadienne. 1997;38(11):703–6. 9360789 PMC1576818

[4] Heimer J, Staudacher O, Steiner F, Kayonga Y, Havugimana JM, Musemakweri A, et al Age-dependent decline and association with stunting of Giardia duodenalis infection among schoolchildren in rural Huye district, Rwanda. Acta tropica. 2015;145:17–22. 10.1016/j.actatropica.2015.01.011 . 25683729

[5] Ignatius R, Gahutu JB, Klotz C, Steininger C, Shyirambere C, Lyng M, et al High prevalence of Giardia duodenalis Assemblage B infection and association with underweight in Rwandan children. PLoS neglected tropical diseases. 2012;6(6):e1677 10.1371/journal.pntd.0001677 22720102 PMC3373622

[6] Olson ME, McAllister TA, Deselliers L, Morck DW, Cheng KJ, Buret AG, et al Effects of giardiasis on production in a domestic ruminant (lamb) model. American journal of veterinary research. 1995;56(11):1470–4. . 8585658

[7] Halliez MC, Buret AG. Extra-intestinal and long term consequences of Giardia duodenalis infections. World journal of gastroenterology: WJG. 2013;19(47):8974–85. 10.3748/wjg.v19.i47.8974 24379622 PMC3870550

[8] Wensaas KA, Langeland N, Hanevik K, Morch K, Eide GE, Rortveit G. Irritable bowel syndrome and chronic fatigue 3 years after acute giardiasis: historic cohort study. Gut. 2012;61(2):214–9. 10.1136/gutjnl-2011-300220 . 21911849

[9] Savioli L, Smith H, Thompson A. Giardia and Cryptosporidium join the 'Neglected Diseases Initiative'. Trends in parasitology. 2006;22(5):203–8. 10.1016/j.pt.2006.02.015 . 16545611

[10] Cotton JA, Beatty JK, Buret AG. Host parasite interactions and pathophysiology in Giardia infections. International journal for parasitology. 2011;41(9):925–33. 10.1016/j.ijpara.2011.05.002 . 21683702

[11] Caccio SM, Ryan U. Molecular epidemiology of giardiasis. Molecular and biochemical parasitology. 2008;160(2):75–80. 10.1016/j.molbiopara.2008.04.006 . 18501440

[12] Lasek-Nesselquist E, Welch DM, Sogin ML. The identification of a new Giardia duodenalis assemblage in marine vertebrates and a preliminary analysis of G. duodenalis population biology in marine systems. International journal for parasitology. 2010;40(9):1063–74. 10.1016/j.ijpara.2010.02.015 20361967 PMC2900473

[13] Xu F, Jerlstrom-Hultqvist J, Andersson JO. Genome-wide analyses of recombination suggest that Giardia intestinalis assemblages represent different species. Molecular biology and evolution. 2012;29(10):2895–8. 10.1093/molbev/mss107 . 22474166

[14] Franzen O, Jerlstrom-Hultqvist J, Castro E, Sherwood E, Ankarklev J, Reiner DS, et al Draft genome sequencing of giardia intestinalis assemblage B isolate GS: is human giardiasis caused by two different species? PLoS pathogens. 2009;5(8):e1000560 10.1371/journal.ppat.1000560 19696920 PMC2723961

[15] Palm D, Weiland M, McArthur AG, Winiecka-Krusnell J, Cipriano MJ, Birkeland SR, et al Developmental changes in the adhesive disk during Giardia differentiation. Molecular and biochemical parasitology. 2005;141(2):199–207. 10.1016/j.molbiopara.2005.03.005 . 15850703

[16] Adam RD. Biology of *Giardia lamblia* . Clinical microbiology reviews. 2001;14(3):447–75. 10.1128/CMR.14.3.447-475.2001 11432808 PMC88984

[17] Ringqvist E, Palm JE, Skarin H, Hehl AB, Weiland M, Davids BJ, et al Release of metabolic enzymes by Giardia in response to interaction with intestinal epithelial cells. Molecular and biochemical parasitology. 2008;159(2):85–91. 10.1016/j.molbiopara.2008.02.005 18359106 PMC3658456

[18] Eckmann L, Laurent F, Langford TD, Hetsko ML, Smith JR, Kagnoff MF, et al Nitric oxide production by human intestinal epithelial cells and competition for arginine as potential determinants of host defense against the lumen-dwelling pathogen Giardia lamblia. Journal of immunology. 2000;164(3):1478–87. . 10640765 10.4049/jimmunol.164.3.1478

[19] Stadelmann B, Hanevik K, Andersson MK, Bruserud O, Svard SG. The role of arginine and arginine-metabolizing enzymes during Giardia—host cell interactions in vitro. BMC microbiology. 2013;13:256 10.1186/1471-2180-13-256 24228819 PMC4225669

[20] Stadelmann B, Merino MC, Persson L, Svard SG. Arginine consumption by the intestinal parasite Giardia intestinalis reduces proliferation of intestinal epithelial cells. PloS one. 2012;7(9):e45325 10.1371/journal.pone.0045325 23028934 PMC3446895

[21] Turk V, Stoka V, Vasiljeva O, Renko M, Sun T, Turk B, et al Cysteine cathepsins: from structure, function and regulation to new frontiers. Biochimica et biophysica acta. 2012;1824(1):68–88. 10.1016/j.bbapap.2011.10.002 . 22024571 PMC7105208

[22] McKerrow JH, Caffrey C, Kelly B, Loke P, Sajid M. Proteases in parasitic diseases. Annual review of pathology. 2006;1:497–536. 10.1146/annurev.pathol.1.110304.100151 . 18039124

[23] Sajid M, McKerrow JH. Cysteine proteases of parasitic organisms. Molecular and biochemical parasitology. 2002;120(1):1–21. . 11849701 10.1016/s0166-6851(01)00438-8

[24] Somanna A, Mundodi V, Gedamu L. Functional analysis of cathepsin B-like cysteine proteases from Leishmania donovani complex. Evidence for the activation of latent transforming growth factor beta. The Journal of biological chemistry. 2002;277(28):25305–12. 10.1074/jbc.M203034200 . 12000761

[25] Kissoon-Singh V, Mortimer L, Chadee K. Entamoeba histolytica cathepsin-like enzymes: interactions with the host gut. Advances in experimental medicine and biology. 2011;712:62–83. 10.1007/978-1-4419-8414-2_5 . 21660659

[26] Aurrecoechea C, Brestelli J, Brunk BP, Carlton JM, Dommer J, Fischer S, et al GiardiaDB and TrichDB: integrated genomic resources for the eukaryotic protist pathogens Giardia lamblia and Trichomonas vaginalis. Nucleic acids research. 2009;37(Database issue):D526–30. 10.1093/nar/gkn631 18824479 PMC2686445

[27] DuBois KN, Abodeely M, Sajid M, Engel JC, McKerrow JH. Giardia lamblia cysteine proteases. Parasitology research. 2006;99(4):313–6. 10.1007/s00436-006-0149-4 . 16598471

[28] Williams AG, Coombs GH. Multiple protease activities in Giardia intestinalis trophozoites. International journal for parasitology. 1995;25(7):771–8. . 7558562 10.1016/0020-7519(94)00201-x

[29] Rodriguez-Fuentes GB, Cedillo-Rivera R, Fonseca-Linan R, Arguello-Garcia R, Munoz O, Ortega-Pierres G, et al Giardia duodenalis: analysis of secreted proteases upon trophozoite-epithelial cell interaction in vitro. Memorias do Instituto Oswaldo Cruz. 2006;101(6):693–6. . 17072486 10.1590/s0074-02762006000600020

[30] Coradi ST, Guimaraes S. Giardia duodenalis: protein substrates degradation by trophozoite proteases. Parasitology research. 2006;99(2):131–6. 10.1007/s00436-005-0124-5 . 16521040

[31] de Carvalho TB, David EB, Coradi ST, Guimaraes S. Protease activity in extracellular products secreted in vitro by trophozoites of Giardia duodenalis. Parasitology research. 2008;104(1):185–90. 10.1007/s00436-008-1185-z . 18797927

[32] Ward W, Alvarado L, Rawlings ND, Engel JC, Franklin C, McKerrow JH. A primitive enzyme for a primitive cell: the protease required for excystation of Giardia. Cell. 1997;89(3):437–44. . 9150143 10.1016/s0092-8674(00)80224-x

[33] DuBois KN, Abodeely M, Sakanari J, Craik CS, Lee M, McKerrow JH, et al Identification of the major cysteine protease of Giardia and its role in encystation. The Journal of biological chemistry. 2008;283(26):18024–31. 10.1074/jbc.M802133200 18445589 PMC2440617

[34] Cotton JA, Bhargava A, Ferraz JG, Yates RM, Beck PL, Buret AG. Giardia duodenalis cathepsin B proteases degrade intestinal epithelial interleukin-8 and attenuate interleukin-8-induced neutrophil chemotaxis. Infection and immunity. 2014 10.1128/IAI.01771-14 . 24733096 PMC4097641

[35] Busatti HG, Santos JF, Gomes MA. The old and new therapeutic approaches to the treatment of giardiasis: where are we? Biologics: targets & therapy. 2009;3:273–87. 19707415 PMC2726062

[36] Turner JR. Intestinal mucosal barrier function in health and disease. Nature reviews Immunology. 2009;9(11):799–809. 10.1038/nri2653 . 19855405

[37] Chin AC, Teoh DA, Scott KG, Meddings JB, Macnaughton WK, Buret AG. Strain-dependent induction of enterocyte apoptosis by Giardia lamblia disrupts epithelial barrier function in a caspase-3-dependent manner. Infection and immunity. 2002;70(7):3673–80. 12065509 10.1128/IAI.70.7.3673-3680.2002 PMC128105

[38] Scott KG, Meddings JB, Kirk DR, Lees-Miller SP, Buret AG. Intestinal infection with Giardia spp. reduces epithelial barrier function in a myosin light chain kinase-dependent fashion. Gastroenterology. 2002;123(4):1179–90. . 12360480 10.1053/gast.2002.36002

[39] Teoh DA, Kamieniecki D, Pang G, Buret AG. Giardia lamblia rearranges F-actin and alpha-actinin in human colonic and duodenal monolayers and reduces transepithelial electrical resistance. The Journal of parasitology. 2000;86(4):800–6. 10.1645/0022-3395(2000)086[0800:GLRFAA]2.0.CO;2 . 10958459

[40] Koh WH, Geurden T, Paget T, O'Handley R, Steuart RF, Thompson RC, et al Giardia duodenalis assemblage-specific induction of apoptosis and tight junction disruption in human intestinal epithelial cells: effects of mixed infections. The Journal of parasitology. 2013;99(2):353–8. 10.1645/GE-3021.1 . 22924932

[41] Costa de Beauregard MA, Pringault E, Robine S, Louvard D. Suppression of villin expression by antisense RNA impairs brush border assembly in polarized epithelial intestinal cells. The EMBO journal. 1995;14(3):409–21. 7859732 10.1002/j.1460-2075.1995.tb07017.x PMC398099

[42] Khurana S, George SP. Regulation of cell structure and function by actin-binding proteins: villin's perspective. FEBS letters. 2008;582(14):2128–39. 10.1016/j.febslet.2008.02.040 18307996 PMC2680319

[43] Buret A, Hardin JA, Olson ME, Gall DG. Pathophysiology of small intestinal malabsorption in gerbils infected with Giardia lamblia. Gastroenterology. 1992;103(2):506–13. . 1634068 10.1016/0016-5085(92)90840-u

[44] Troeger H, Epple HJ, Schneider T, Wahnschaffe U, Ullrich R, Burchard GD, et al Effect of chronic Giardia lamblia infection on epithelial transport and barrier function in human duodenum. Gut. 2007;56(3):328–35. 10.1136/gut.2006.100198 16935925 PMC1856804

[45] Wang Y, Srinivasan K, Siddiqui MR, George SP, Tomar A, Khurana S. A novel role for villin in intestinal epithelial cell survival and homeostasis. The Journal of biological chemistry. 2008;283(14):9454–64. 10.1074/jbc.M707962200 . 18198174

[46] Scott KG, Yu LC, Buret AG. Role of CD8+ and CD4+ T lymphocytes in jejunal mucosal injury during murine giardiasis. Infection and immunity. 2004;72(6):3536–42. 10.1128/IAI.72.6.3536-3542.2004 15155662 PMC415705

[47] Tomar A, George S, Kansal P, Wang Y, Khurana S. Interaction of phospholipase C-gamma1 with villin regulates epithelial cell migration. The Journal of biological chemistry. 2006;281(42):31972–86. 10.1074/jbc.M604323200 . 16921170

[48] Wang Y, Tomar A, George SP, Khurana S. Obligatory role for phospholipase C-gamma(1) in villin-induced epithelial cell migration. American journal of physiology Cell physiology. 2007;292(5):C1775–86. 10.1152/ajpcell.00420.2006 . 17229814

[49] Athman R, Louvard D, Robine S. The epithelial cell cytoskeleton and intracellular trafficking. III. How is villin involved in the actin cytoskeleton dynamics in intestinal cells? American journal of physiology Gastrointestinal and liver physiology. 2002;283(3):G496–502. 10.1152/ajpgi.00207.2002 . 12181160

[50] Athman R, Louvard D, Robine S. Villin enhances hepatocyte growth factor-induced actin cytoskeleton remodeling in epithelial cells. Molecular biology of the cell. 2003;14(11):4641–53. 10.1091/mbc.E03-02-0091 12937273 PMC266779

[51] Solaymani-Mohammadi S, Singer SM. Regulation of intestinal epithelial cell cytoskeletal remodeling by cellular immunity following gut infection. Mucosal immunology. 2013;6(2):369–78. 10.1038/mi.2012.80 . 22910215 PMC4094376

[52] Barrett AJ, Kembhavi AA, Brown MA, Kirschke H, Knight CG, Tamai M, et al L-trans-Epoxysuccinyl-leucylamido(4-guanidino)butane (E-64) and its analogues as inhibitors of cysteine proteinases including cathepsins B, H and L. The Biochemical journal. 1982;201(1):189–98. 7044372 10.1042/bj2010189 PMC1163625

[53] Tchoupe JR, Moreau T, Gauthier F, Bieth JG. Photometric or fluorometric assay of cathepsin B, L and H and papain using substrates with an aminotrifluoromethylcoumarin leaving group. Biochimica et biophysica acta. 1991;1076(1):149–51. . 1986788 10.1016/0167-4838(91)90232-o

[54] Barrett AJ. Fluorimetric assays for cathepsin B and cathepsin H with methylcoumarylamide substrates. The Biochemical journal. 1980;187(3):909–12. 6897924 10.1042/bj1870909 PMC1162479

[55] Musil D, Zucic D, Turk D, Engh RA, Mayr I, Huber R, et al The refined 2.15 A X-ray crystal structure of human liver cathepsin B: the structural basis for its specificity. The EMBO journal. 1991;10(9):2321–30. 1868826 10.1002/j.1460-2075.1991.tb07771.x PMC452927

[56] Favennec L, Chochillon C, Meillet D, Magne D, Savel J, Raichvarg D, et al Adherence and multiplication of Giardia intestinalis on human enterocyte-like differentiated cells in vitro. Parasitology research. 1990;76(7):581–4. . 2217120 10.1007/BF00932566

[57] Muller J, Ruhle G, Muller N, Rossignol JF, Hemphill A. In vitro effects of thiazolides on Giardia lamblia WB clone C6 cultured axenically and in coculture with Caco2 cells. Antimicrobial agents and chemotherapy. 2006;50(1):162–70. 10.1128/AAC.50.1.162-170.2006 16377682 PMC1346829

[58] Buret A, denHollander N, Wallis PM, Befus D, Olson ME. Zoonotic potential of giardiasis in domestic ruminants. The Journal of infectious diseases. 1990;162(1):231–7. . 2355197 10.1093/infdis/162.1.231

[59] Aggarwal A, Merritt JW Jr., Nash TE. Cysteine-rich variant surface proteins of Giardia lamblia. Molecular and biochemical parasitology. 1989;32(1):39–47. . 2911277 10.1016/0166-6851(89)90127-8

[60] Diamond LS, Harlow DR, Cunnick CC. A new medium for the axenic cultivation of Entamoeba histolytica and other Entamoeba. Transactions of the Royal Society of Tropical Medicine and Hygiene. 1978;72(4):431–2. . 212851 10.1016/0035-9203(78)90144-x

[61] Keister DB. Axenic culture of Giardia lamblia in TYI-S-33 medium supplemented with bile. Transactions of the Royal Society of Tropical Medicine and Hygiene. 1983;77(4):487–8. . 6636276 10.1016/0035-9203(83)90120-7

[62] Coklin T, Farber J, Parrington L, Dixon B. Prevalence and molecular characterization of Giardia duodenalis and Cryptosporidium spp. in dairy cattle in Ontario, Canada. Vet Parasitol. 2007;150(4):297–305. 10.1016/j.vetpar.2007.09.014 . 17964724

[63] Read CM, Monis PT, Thompson RC. Discrimination of all genotypes of Giardia duodenalis at the glutamate dehydrogenase locus using PCR-RFLP. Infect Genet Evol. 2004;4(2):125–30. 10.1016/j.meegid.2004.02.001 . 15157630

[64] Hall TA, editor BioEdit: a user-friendly biological sequence alignment editor and analysis program for Windows 95/98/NT. Nucleic acids symposium series; 1999.

[65] Upcroft JA, Upcroft P. Drug susceptibility testing of anaerobic protozoa. Antimicrobial agents and chemotherapy. 2001;45(6):1810–4. 10.1128/AAC.45.6.1810-1814.2001 11353630 PMC90550

[66] Bradford MM. A rapid and sensitive method for the quantitation of microgram quantities of protein utilizing the principle of protein-dye binding. Analytical biochemistry. 1976;72:248–54. . 942051 10.1016/0003-2697(76)90527-3

[67] Carvalho TB, Oliveira-Sequeira TC, Guimaraes S. In vitro ANTIGIARDIAL ACTIVITY OF THE CYSTEINE PROTEASE INHIBITOR E-64. Revista do Instituto de Medicina Tropical de Sao Paulo. 2014;56(1):43–7. 10.1590/S0036-46652014000100006 . 24553607 PMC4085827

[68] Lauwaet T, Andersen Y, Van de Ven L, Eckmann L, Gillin FD. Rapid detachment of Giardia lamblia trophozoites as a mechanism of antimicrobial action of the isoflavone formononetin. The Journal of antimicrobial chemotherapy. 2010;65(3):531–4. 10.1093/jac/dkp501 20067984 PMC2818108

[69] Buret AG, Mitchell K, Muench DG, Scott KG. Giardia lamblia disrupts tight junctional ZO-1 and increases permeability in non-transformed human small intestinal epithelial monolayers: effects of epidermal growth factor. Parasitology. 2002;125(Pt 1):11–9. . 12166516 10.1017/s0031182002001853

[70] Lauwaet T, Oliveira MJ, Callewaert B, De Bruyne G, Saelens X, Ankri S, et al Proteolysis of enteric cell villin by Entamoeba histolytica cysteine proteinases. The Journal of biological chemistry. 2003;278(25):22650–6. 10.1074/jbc.M300142200 . 12690119

[71] Humen MA, Perez PF, Lievin-Le Moal V. Lipid raft-dependent adhesion of Giardia intestinalis trophozoites to a cultured human enterocyte-like Caco-2/TC7 cell monolayer leads to cytoskeleton-dependent functional injuries. Cellular microbiology. 2011;13(11):1683–702. 10.1111/j.1462-5822.2011.01647.x . 21790940

[72] Ringqvist E, Avesson L, Soderbom F, Svard SG. Transcriptional changes in Giardia during host-parasite interactions. International journal for parasitology. 2011;41(3–4):277–85. 10.1016/j.ijpara.2010.09.011 . 21074536

[73] Moncada D, Keller K, Ankri S, Mirelman D, Chadee K. Antisense inhibition of Entamoeba histolytica cysteine proteases inhibits colonic mucus degradation. Gastroenterology. 2006;130(3):721–30. 10.1053/j.gastro.2005.11.012 . 16530514

[74] Carabeo RA, Grieshaber SS, Fischer E, Hackstadt T. Chlamydia trachomatis induces remodeling of the actin cytoskeleton during attachment and entry into HeLa cells. Infection and immunity. 2002;70(7):3793–803. 12065523 10.1128/IAI.70.7.3793-3803.2002 PMC128046

[75] Walker ME, Hjort EE, Smith SS, Tripathi A, Hornick JE, Hinchcliffe EH, et al Toxoplasma gondii actively remodels the microtubule network in host cells. Microbes and infection / Institut Pasteur. 2008;10(14–15):1440–9. 10.1016/j.micinf.2008.08.014 18983931 PMC2765197

[76] Yang W, McCrae MA. The rotavirus enterotoxin (NSP4) promotes re-modeling of the intracellular microtubule network. Virus research. 2012;163(1):269–74. 10.1016/j.virusres.2011.10.011 . 22036730

[77] Elliott DA, Clark DP. Cryptosporidium parvum induces host cell actin accumulation at the host-parasite interface. Infection and immunity. 2000;68(4):2315–22. 10722635 10.1128/iai.68.4.2315-2322.2000 PMC97419

[78] Battle SE, Brady MJ, Vanaja SK, Leong JM, Hecht GA. Actin pedestal formation by enterohemorrhagic Escherichia coli enhances bacterial host cell attachment and concomitant type III translocation. Infection and immunity. 2014;82(9):3713–22. 10.1128/IAI.01523-13 24958711 PMC4187837

[79] Bonnin A, Lapillonne A, Petrella T, Lopez J, Chaponnier C, Gabbiani G, et al Immunodetection of the microvillous cytoskeleton molecules villin and ezrin in the parasitophorous vacuole wall of Cryptosporidium parvum (Protozoa: Apicomplexa). European journal of cell biology. 1999;78(11):794–801. 10.1016/S0171-9335(99)80030-2 . 10604656

[80] Lhocine N, Arena ET, Bomme P, Ubelmann F, Prevost MC, Robine S, et al Apical Invasion of Intestinal Epithelial Cells by Salmonella typhimurium Requires Villin to Remodel the Brush Border Actin Cytoskeleton. Cell host & microbe. 2015;17(2):164–77. 10.1016/j.chom.2014.12.003 25600187 PMC4346658

[81] Athman R, Fernandez MI, Gounon P, Sansonetti P, Louvard D, Philpott D, et al Shigella flexneri infection is dependent on villin in the mouse intestine and in primary cultures of intestinal epithelial cells. Cellular microbiology. 2005;7(8):1109–16. 10.1111/j.1462-5822.2005.00535.x . 16008578

[82] Finlay BB, Falkow S. Salmonella interactions with polarized human intestinal Caco-2 epithelial cells. The Journal of infectious diseases. 1990;162(5):1096–106. . 2230236 10.1093/infdis/162.5.1096

[83] Goosney DL, Knoechel DG, Finlay BB. Enteropathogenic E. coli, Salmonella, and Shigella: masters of host cell cytoskeletal exploitation. Emerging infectious diseases. 1999;5(2):216–23. 10.3201/eid0502.990205 10221873 PMC2640686

[84] Krumkamp R, Sarpong N, Schwarz NG, Adelkofer J, Loag W, Eibach D, et al Gastrointestinal infections and diarrheal disease in ghanaian infants and children: an outpatient case-control study. PLoS neglected tropical diseases. 2015;9(3):e0003568 10.1371/journal.pntd.0003568 25738935 PMC4349824

[85] Wang L, Xiao L, Duan L, Ye J, Guo Y, Guo M, et al Concurrent infections of Giardia duodenalis, Enterocytozoon bieneusi, and Clostridium difficile in children during a cryptosporidiosis outbreak in a pediatric hospital in China. PLoS neglected tropical diseases. 2013;7(9):e2437 10.1371/journal.pntd.0002437 24069491 PMC3772047

[86] Ankarklev J, Hestvik E, Lebbad M, Lindh J, Kaddu-Mulindwa DH, Andersson JO, et al Common coinfections of Giardia intestinalis and Helicobacter pylori in non-symptomatic Ugandan children. PLoS neglected tropical diseases. 2012;6(8):e1780 10.1371/journal.pntd.0001780 22953010 PMC3429385

[87] Bilenko N, Levy A, Dagan R, Deckelbaum RJ, El-On Y, Fraser D. Does co-infection with Giardia lamblia modulate the clinical characteristics of enteric infections in young children? European journal of epidemiology. 2004;19(9):877–83. . 15499898 10.1023/b:ejep.0000040533.75646.9c

[88] Veenemans J, Mank T, Ottenhof M, Baidjoe A, Mbugi EV, Demir AY, et al Protection against diarrhea associated with Giardia intestinalis Is lost with multi-nutrient supplementation: a study in Tanzanian children. PLoS neglected tropical diseases. 2011;5(6):e1158 10.1371/journal.pntd.0001158 21666789 PMC3110167

[89] Avila EE, Calderon J. Entamoeba histolytica trophozoites: a surface-associated cysteine protease. Experimental parasitology. 1993;76(3):232–41. 10.1006/expr.1993.1028 . 8500583

[90] Jacobs T, Bruchhaus I, Dandekar T, Tannich E, Leippe M. Isolation and molecular characterization of a surface-bound proteinase of Entamoeba histolytica. Molecular microbiology. 1998;27(2):269–76. . 9484883 10.1046/j.1365-2958.1998.00662.x

[91] Garcia-Rivera G, Rodriguez MA, Ocadiz R, Martinez-Lopez MC, Arroyo R, Gonzalez-Robles A, et al Entamoeba histolytica: a novel cysteine protease and an adhesin form the 112 kDa surface protein. Molecular microbiology. 1999;33(3):556–68. . 10417646 10.1046/j.1365-2958.1999.01500.x

[92] Lidell ME, Moncada DM, Chadee K, Hansson GC. Entamoeba histolytica cysteine proteases cleave the MUC2 mucin in its C-terminal domain and dissolve the protective colonic mucus gel. Proceedings of the National Academy of Sciences of the United States of America. 2006;103(24):9298–303. 10.1073/pnas.0600623103 16754877 PMC1482604

